# Baseline-dependent network reactivity to visual input in children with autism spectrum disorder: a magnetoencephalography study

**DOI:** 10.3389/fpsyt.2025.1600973

**Published:** 2025-07-16

**Authors:** Tetsu Hirosawa, Daiki Soma, Masuhiko Sano, Masafumi Kameya, Yuko Yoshimura, Sumie Iwasaki, Sanae Tanaka, Mitsuru Kikuchi

**Affiliations:** ^1^ Department of Psychiatry and Neurobiology, Graduate School of Medical Science, Kanazawa University, Kanazawa, Japan; ^2^ Research Center for Child Mental Development, Kanazawa University, Kanazawa, Japan; ^3^ Faculty of Education, Institute of Human and Social Sciences, Kanazawa University, Kanazawa, Japan

**Keywords:** autism spectrum disorder, magnetoencephalography, visual stimuli, graph theory, small-worldness, social communication

## Abstract

**Background/aims:**

Neuroimaging studies suggest altered functional brain organization in children with autism spectrum disorder (ASD), particularly in response to visual stimulation. However, how transitions between different visual states modulate brain network in ASD remains unclear. This study aimed to investigate how transitioning from minimal visual input (fixation in a dark room, DR) to a silent video (eyes open, EO) alters functional brain networks in children with ASD compared with their typically developing (TD) peers.

**Methods:**

We analyzed magnetoencephalography (MEG) data from children with ASD (n=23) and TD children (n=31), aged 3–10 years. MEG signals were mapped to 68 cortical regions using the Desikan–Killiany atlas, and functional connectivity was assessed using the phase lag index across five frequency bands (delta, theta, alpha, beta, and gamma). Graph theoretical analyses quantified the clustering coefficient (C), characteristic path length (L), and small-worldness (SW) to evaluate network organization.

**Results:**

Both groups exhibited increased alpha-band clustering coefficients under EO. Notably, baseline (DR) graph metrics predicted EO-induced changes, with higher initial values associated with smaller subsequent increases. Diagnosis-by-condition interactions emerged in the delta and beta bands: children with ASD exhibited more pronounced increases in SW from DR to EO, whereas TD peers showed more modest or opposite shifts. Within the ASD group, larger beta-band SW increases correlated with greater autistic trait severity (Social Responsiveness Scale), whereas in TD children, delta-band increases associated with milder autistic-like traits.

**Conclusion:**

These findings reveal age- and diagnosis-specific differences in how visual stimulation reshapes functional brain network organization. They also highlight the potential of network metrics as biomarkers for ASD, though validation in larger, more diverse cohorts is needed to establish clinical relevance.

## 1 Introduction

Autism spectrum disorder (ASD) is a neurodevelopmental condition characterized by impairments in social interaction and communication, as well as restricted, repetitive behaviors and fixated interests (1). Diagnosing ASD can be challenging owing to the subtlety and variability of behavioral markers, time constraints during clinical evaluations, and comorbidities such as anxiety or hyperactivity. Additional factors, including female sex, mild symptom presentation, inconsistent care, socioeconomic status, and language barriers, may further obscure or delay an accurate diagnosis (2–5). Given these complexities, investigating the biological and physiological underpinnings of ASD may improve diagnostic precision. In recent years, brain imaging techniques have become essential tools for exploring the neural basis of ASD (6). Electroencephalography (EEG) and magnetoencephalography (MEG) provide insights into neural processing by measuring the brain’s electrical and magnetic activity, respectively. Both techniques are safe, noninvasive, and free from noise or radiation, making them well-suited for pediatric research.

The recent systematic review and meta‐analysis by Neo et al. (7) have highlighted atypical patterns of resting‐state EEG in ASD, underscoring the influence of recording conditions—eyes open (EO) versus eyes closed or EO with minimal visual input (EC)—on observed group differences. Specifically, EO paradigms tend to reveal more robust ASD‐related deviations than EC paradigms. Likewise, Mathewson et al. (8) demonstrated that alpha suppression—a hallmark of cortical reactivity to visual input—is attenuated in ASD. Together, these findings emphasize the importance of distinguishing EO and EC states when investigating atypical brain oscillations in ASD and suggest that directly contrasting these conditions may shed light on the neurophysiological mechanisms underlying this disorder.

Given the brain’s inherent complexity, relying solely on simple measures, such as power spectra, may oversimplify its dynamics. To address this, neuroscience has increasingly adopted graph theory as a framework for understanding large-scale brain networks (9). Within this framework, a complex system is represented as a “graph” consisting of nodes (discrete objects) and edges (relationships between them). In brain network models, nodes correspond to distinct brain regions, whereas edges reflect their functional connectivity (10). Key metrics such as the mean clustering coefficient, which quantifies local clustering among connected nodes (functional segregation), and the average shortest path length, which reflects communication efficiency across distant regions (functional integration), help characterize network organization (11). In healthy brains, high clustering and short path lengths typically coexist, forming a “small-world” configuration that optimally balances local specialization and global integration (12–16). This property, known as “small-worldness”, is measured by the ratio of normalized clustering to normalized path length, highlighting the brain’s efficient information-processing architecture.

Given that simple spectral metrics—such as alpha power and peak alpha frequency (7, 8)—already differ between EO and EC resting-state EEGs, it is essential to treat these states separately in graph-theoretical analyses. Although many EEG and MEG studies have used graph theory to characterize functional networks in ASD, none have specifically compared EO with EC. To the best of our knowledge, even only a few studies have examined both states in healthy adults (17–19). In these cohorts, clustering coefficients are generally higher and characteristic path lengths are longer in the alpha band under EC compared to EO conditions (17, 18), with the sole exception of Zheng et al. (19), who reported the reverse pattern for path length. Although these findings derive from non-ASD populations, they clearly demonstrate that EO–EC manipulations modulate key graph metrics, underscoring the importance of distinguishing these states in graph-theoretical research.

Beyond EC–EO distinctions, the choice of functional-connectivity metric critically shapes graph-theoretical outcomes, as different measures rest on distinct assumptions and may yield substantially different connectivity estimates (20). Although some studies suggest that broad patterns of connectivity may converge across methods (21), the magnitude and topological properties of resulting networks can still differ considerably. In graph-theoretical analyses, connectivity measures that are highly vulnerable to volume conduction and source leakage—such as correlation, coherence, and synchronization likelihood—are generally discouraged, as spatial leakage can introduce strong but spurious connections (20, 21), leading to inflated clustering coefficients and other distorted network metrics. A common strategy to address this issue is to use phase-based connectivity measures that specifically suppress zero-phase interactions, which are most susceptible to volume conduction. While this comes at the cost of discarding genuine zero-lag synchrony, it greatly improves the physiological plausibility of the estimated networks. Among such measures, the phase lag index (PLI) exhibits high test–retest reliability (22) and is widely used in pediatric ASD studies employing EEG or MEG (22–27).

Network construction is another key methodological concern that must be considered. Functional brain networks in graph theory are built upon the estimated strength of functional connectivity between brain regions (nodes). In a weighted network, these connection strengths are retained, whereas in an unweighted (binary) network, only the presence or absence of a connection is considered. Binary networks are typically constructed by applying a threshold to the connectivity matrix, such that connections above this threshold are retained and others are discarded. One rationale for using binary networks is to eliminate potentially spurious connections, which may arise from noise or methodological artifacts (10). However, threshold selection is often arbitrary and can vary across research groups, potentially biasing results (28). Weighted networks avoid the need for thresholding and offer a more continuous, realistic representation of functional brain organization. Nevertheless, they include all connections—including weak ones—that may be dominated by noise, thereby affecting graph metrics. Furthermore, networks can be either directed, reflecting the directionality of interactions (typically based on effective connectivity), or undirected, which assumes symmetric relationships. While directed networks can potentially yield deeper insights into causal information flow, their interpretation is more complex and depends on additional modeling assumptions. Overall, the information carried by graphs ranges from the simplest (binary and undirected) to the most detailed (weighted and directed), with richer representations often coming at the expense of interpretability and robustness. Possibly reflecting this trade-off, prior studies in children with and without ASD have used a range of approaches: binary undirected graphs (23, 26, 27, 29), weighted undirected graphs (24, 25, 30), and rarely binary directed graphs (31).

Finally, two common EEG-specific concerns in graph-theoretical analyses must be addressed: reference montage and sensor density. Unlike MEG, EEG potentials are measured relative to a chosen reference—often a single electrode (e.g., mastoid, earlobe, or central)—or in bipolar configurations. Single-reference montages inherently include reference-site activity, which can confound connectivity estimates. Although re-referencing (e.g., average reference) can reduce this contamination (32), different referencing methods yield distinct sensor-level connectivity patterns (33), and single-reference montages introduce greater distortions than average referencing (34). Reference choice also significantly impacts graph metrics—such as node degree and local efficiency—derived from these estimates (34). Given ongoing debates regarding the optimal reference for connectivity and graph-theoretical measures (20), caution is warranted when interpreting EEG-based networks. Electrode density similarly affects network topology. Hatlestad-Hall et al. (35) showed that reducing the electrode count leads to an overestimation of clustering coefficients and an underestimation of characteristic path length; these authors recommend at least 64 sensors for reliable graph metrics.

Several EEG and MEG studies have applied graph-theoretical methods to compare functional brain networks between ASD and typically developing (TD) groups (23, 25–27, 29–31). However, methodological variability—including differences in recording conditions (EO vs. EC), connectivity metrics (e.g., coherence vs. PLI), graph construction (binary vs. weighted networks), and participant characteristics (age, medication status)—has resulted in inconsistent findings. For example, some studies have reported reduced small-worldness in certain frequency bands of the ASD group (23, 25, 29), whereas others have found enhanced small-worldness (26, 30). These discrepancies highlight the importance of explicitly considering methodological differences when interpreting graph-theoretical findings in ASD. Focusing specifically on pediatric studies employing PLI-based binary undirected graphs—as in the present study—only three prior studies meet these criteria (23, 26, 27), and all three studies used MEG. Soma et al. (23) examined young children (60–89 months) under minimal visual input (fixation cross), reporting reduced small-worldness in the beta band in ASD, alongside a negative correlation between beta-band small-worldness and autistic traits measured by Social Responsiveness Scale (SRS) scores. In contrast, two studies employing rich visual inputs (e.g., video viewing) yielded mixed results. Shiota et al. (27) studied children aged 38–92 months, grouped them by autistic trait severity, and reported reduced small-worldness across multiple frequency bands (delta, theta, beta, and gamma) in children with pronounced autistic traits. Takahashi et al. (26), however, found increased gamma-band small-worldness and decreased delta-band small-worldness in ASD. These conflicting results likely stem from methodological disparities: Takahashi et al. conducted sensor-level analyses and included medicated participants, whereas Shiota et al. conducted source-level analyses and excluded participants on medication. Given the methodological similarities (source-level analysis, unmedicated participants) between Soma et al. (23) and Shiota et al. (27), the findings from these two studies may offer more reliable comparative insights. Taken together, these pediatric findings suggest that ASD is generally characterized by reduced small-worldness under conditions of robust visual stimulation (27), whereas under minimal visual input, the observed differences are largely confined to the beta band (23).

The primary objective of this study was to determine how transitions from minimal visual stimulation (dark room (DR)) to robust visual stimulation (eyes open (EO)) influence three critical graph metrics—small-worldness, normalized clustering coefficient, and normalized characteristic path length—in children with and without ASD. Given the methodological variability in the currently available literature, we adopted a PLI-based connectivity measure to reduce sensitivity to spurious connections from volume conduction and to enhance comparability with prior pediatric ASD studies. Furthermore, we used binary undirected graphs, aligning with the dominant approach in the pediatric literature to balance methodological consistency, interpretability, and robustness. To overcome challenges associated with EEG reference-montage confounds and electrode density limitations, we utilized a child-specific, 151-channel MEG system. MEGs measure unreferenced magnetic fields, thereby avoiding EEG-specific reference distortions. Additionally, the high sensor density and pediatric-optimized helmet design improve signal-to-noise ratios, enhance spatial sampling, and provide more accurate source-level estimates of connectivity and network organization.

Building upon the findings by Soma et al. (23) and Shiota et al. (27), we expected condition-dependent differences in small-world network properties between ASD and TD groups. Specifically, we hypothesized that during the transition from DR to EO conditions, children with ASD would exhibit reduced reactivity—particularly diminished small-worldness changes—in delta, theta, and gamma bands compared to their TD peers. For completeness, we also examined whether similar effects would emerge for the characteristic path length L and the clustering coefficient C across the same frequency bands. As additional exploratory analyses, we investigated whether autistic traits were associated with EO-induced network changes and whether baseline DR measures predicted the magnitude of these dynamic shifts.

## 2 Materials and methods

### 2.1 Study design and participants

In this prospective observational study, we recruited children aged 3–10 years with ASD and TD peers for MEG recordings.

The ASD group consisted of 39 children diagnosed with ASD, recruited from Kanazawa University and its affiliated hospitals. Only those with a confirmed ASD diagnosis without any additional neuropsychiatric conditions (e.g., attention-deficit/hyperactivity disorder, anxiety, epilepsy) were included, as verified by the referring clinician. The ASD diagnoses were further confirmed using either the Diagnostic Interview for Social and Communication Disorders (DISCO) or the Autism Diagnostic Observation Schedule-2 (ADOS-2) (1, 36–38). TD children were recruited through flyers and an institutional website. Inclusion criteria were the absence of known developmental, psychiatric, or neurological diagnoses based on parental reports. The control group included 62 TD children with no known behavioral or language difficulties. Participants currently taking psychotropic or neurological medications were excluded from both groups. If a medication was initiated after recruitment but before the MEG session, participants were instructed to pause this medication for at least 24 h before scanning. Information on past medication history was not systematically collected.

Children were also excluded if they met any of the following criteria: (1) sensory impairments (blindness or deafness), (2) intellectual disabilities (see Section 2.2 for details), or (3) incomplete MEG or magnetic resonance imaging (MRI) data. In the final sample, 16 children with ASD and 31 TD children were excluded due to incomplete MEG, magnetic resonance imaging, or psychological assessments (see RESULTS section for details). All participants were of Japanese ethnicity.

Written informed consent was obtained from the parents of the children before participation in the study. The study was approved by the Ethics Committee of Kanazawa University Hospital and conducted in accordance with the Declaration of Helsinki. This research was part of the broader Bambi Plan at the Kanazawa University Research Center for Child Mental Development (https://kodomokokoro.w3.kanazawa-u.ac.jp/en/). While some participants in this study were also included in our previous study (23), there was no overlap in the results, and the objectives of that study differed significantly from those of the present study.

### 2.2 Assessment of intelligence and the severity of autism symptoms

To evaluate intellectual functioning, we administered the Kaufman Assessment Battery for Children (K-ABC) or its second edition (K-ABC-II) to all participants depending on the time of assessment and test availability (39, 40). The K-ABC provides a Mental Processing Scale (MPS) that assesses problem-solving skills through simultaneous and sequential processing tasks, whereas the K-ABC-II offers the Mental Processing Index (MPI), which is conceptually similar to the original K-ABC MPS, measuring general mental processing abilities. As our study focused on children with ASD but without intellectual disabilities, we set an inclusion criterion of a score of ≥70 on these scales. This threshold aligns with the standard diagnostic criteria, distinguishing intellectual disability from average intellectual functioning (1).

To assess the severity of autism symptoms, parents completed the SRS or its second edition (SRS-2) (41, 42). The SRS and SRS-2 provide a continuous measure of social ability, ranging from impaired to above average, rather than a categorical ASD diagnosis. Higher scores were associated with greater social impairment. Because the SRS measures autism traits along a spectrum, it can identify both milder ASD symptoms, as well as social impairments in non-ASD individuals.

### 2.3 MEG data acquisition

MEG data were recorded using a 151-channel Superconducting Quantum Interference Device (SQUID) whole-head coaxial gradiometer system (PQ 1151R; Yokogawa/KIT, Kanazawa, Japan) housed in a magnetically shielded room (Daido Steel Co., Ltd., Nagoya, Japan). To optimize sensor positioning for children’s heads and minimize movement, we used a custom-made child-sized MEG system (43). MEG signals were low-pass filtered at 500 Hz and sampled at 2,000 Hz. Recordings were conducted under two conditions: (1) DR Condition – participants focused on a fixation cross in a dark room, approximating a resting-state condition (consistent with our previous studies) (23, 44), and (2) EO Condition – participants watched a silent video projected onto a screen. During both conditions, participants lay supine on a bed.

To enhance engagement and minimize movement, each child selected a preferred video from a set of popular programs. Given that children with ASD may experience heightened sensory sensitivity, this approach, reduced anxiety by providing a sense of control, minimized movement artifacts, improved data quality, and increased compliance, ultimately leading to higher-quality MEG recordings.

Although this strategy sacrificed consistency in visual stimuli across participants, the benefits of reducing stress and obtaining cleaner recordings outweighed this limitation. A staff member remained in the room to encourage stillness.

Recordings were conducted between 11:00 AM and 3:00 PM. No participant showed clear signs of drowsiness based on MEG waveforms. Given the challenge of keeping young children stationary, we set a minimum recording duration of 50 s consistent with our previous studies (23, 26). To account for data loss due to movement artifacts, we recorded 130 s for the DR condition and 190 s for the EO condition.

These durations ensured sufficient artifact-free data for analysis while maintaining participant comfort.

### 2.4 Magnetic resonance imaging

Structural brain images were acquired using a 1.5 Tesla (T) MRI scanner (SIGNA Explorer; GE Healthcare, USA) with a T1-weighted gradient-echo sequence incorporating the Silenz pulse sequence. This specialized sequence minimizes acoustic noise and shortens scan times, making it particularly suitable for pediatric populations (45, 46). The imaging parameters were as follows: repetition time (TR) = 435.68 ms, echo time (TE) = 0.024 ms, flip angle = 7°, field of view = 220 mm, matrix size = 256 × 256 pixels, and slice thickness = 1.7 mm, yielding a total of 130 transaxial images. Although this protocol resulted in slightly lower spatial resolution due to the thicker slices and lower matrix size, it provided sufficient anatomical reference while minimizing scan duration to enhance participant compliance.

### 2.5 Co-registration of MEG and MRI images

Co-registration of MEG and MRI images was performed using specific anatomical markers. Four key reference points were identified in both modalities: the midline frontal point, vertex, and bilateral mastoid processes. For MEG, magnetic field-generating coils served as markers, while for MRI, lipid capsules were used due to their high-intensity appearance in the images. Additionally, anatomical landmarks such as the mastoid processes, nasion, and skull surface were manually identified on MRI scans. To enhance accuracy, 15–25 additional points were marked for each participant, ensuring precise alignment between MEG and MRI data.

### 2.6 MEG data preprocessing

MEG data were processed using Brainstorm (47), an open-source software platform available under the GNU General Public License. Preprocessing followed the guidelines of the Organization for Human Brain Mapping (48). The preprocessing pipeline was identical to that detailed in Kameya et al. (49) and in our earlier work (23, 26).

The preprocessing pipeline included the following steps:

1. Downsampling: Data were downsampled to 500 Hz to reduce computational load while preserving temporal resolution.
2. Sensor Exclusion: Noisy sensors were identified and excluded.
3. Artifact Removal:
  • Notch filters at 60, 120, and 180 Hz were applied to eliminate power-line noise and harmonics.
  • A 0.5–200 Hz band-pass filter was applied to retain relevant frequency components.
  • Independent component analysis (ICA) was used to remove ocular and cardiac artifacts, specifically targeting blinks and heartbeat-related noise.
4. Manual Inspection: Segments with apparent motion artifacts or radio frequency interference were visually identified and removed by one of the authors (Daiki Soma), who was blinded to participant identities.
5. Epoching: Continuous MEG data were segmented into 5-s epochs. To ensure sufficient data quality, at least 10 artifact-free segments (≥50 s total recording time) were retained per participant.
6. Frequency Band Filtering: Each epoch was further decomposed into the following commonly used frequency bands:
  • Delta (2–4 Hz)
  • Theta (4–8 Hz)
  • Alpha (8–13 Hz)
  • Beta (13–30 Hz)
  • Gamma (30–60 Hz)

### 2.7 Atlas-guided source reconstruction and segmentation

Signal source estimation was performed using each participant’s original anatomical data. An anatomically constrained MEG approach was employed, applying structural constraints to estimated sources (50). Head models were computed using the overlapping spheres algorithm (51) with a default source space comprising 15,000 vertices. We used weighted minimum-norm estimation (wMNE) to determine source orientation constraints (52). Since noise recordings were unavailable, an identity matrix was used as the noise covariance. Signal sources were then grouped into 68 regions based on the Desikan–Killiany atlas (53), utilizing principal component analysis to refine signal grouping.

We selected the Desikan–Killiany atlas to balance the limitations of MEG’s spatial resolution with the need for interpretable results. Choosing an appropriate brain parcellation scheme is critical for graph-theoretical analysis of functional networks. The number of regions of interest (ROIs) must balance interpretability and spatial resolution (54): Fewer ROIs improve interpretability but risk oversimplification by merging functionally distinct areas. More ROIs capture finer connectivity details but increase complexity and potential signal leakage.

While Hallquist and Hillary (55) recommended segmenting the brain into ≥200 functional regions for fMRI studies, MEG’s lower spatial resolution necessitates a more conservative approach. Farahibozorg et al. (56) suggested that approximately 70 parcels optimize spatial resolution while minimizing signal leakage in MEG studies. Based on these findings, we adopted the Desikan–Killiany atlas, which provides a 68-region cortical parcellation, as an optimal balance for our study.

### 2.8 Phase lag index as a connectivity measure

PLI was used to estimate functional connectivity between signal sources by assessing phase relationships in time series signals (56). However, reconstructed sources may contain spurious interactions due to volume conduction or field spread, which can cause artificial synchrony, particularly at zero-lag phase differences (57). To mitigate zero-lag synchrony and focus on meaningful connectivity, we employed PLI, a mixing-insensitive interaction metric that attenuates artificial interactions by emphasizing consistent nonzero phase lags (58).

For each epoch, the instantaneous phase of the filtered waveform was computed using the Hilbert transform for each signal source. The phase difference *Δϕ(t_k_)* was then computed between each pair of sources at each time point *t_k_* (where *k* = 1, 2, 3, …, N, and N is the number of time points per epoch). The PLI between two signal sources in an epoch is defined as follows (58):


$$PLI=\left|\frac{1}{N}\sum_{k=1}^{N}sign[\text{Δ}\varphi (t_{k})]\right|$$

where the sign function of the phase difference at time point “*t_k_*,” is defined as:

- +1 if *Δϕ(t*_k_ *)* > 0
−1 if *Δϕ(t*_k_ *)* < 0
- 0 if *Δϕ(t*_k_ *)* = 0

PLI values range from 0 to 1, inclusive. A value closer to 1 indicates a strong nonzero phase lag between the two signals over time, implying robust phase synchronization, whereas a value near 0 suggests weak or no consistent phase relationship. Notably, PLI does not indicate which of the signal is leads in phase, only the consistency of phase lag. PLI was computed for all signal source pairs across each frequency band to estimate functional connectivity.

### 2.9 Graph construction and graph metrics

To characterize the brain functional connectivity, we employed graph theory, representing the brain network as a graph of nodes and edges. The network comprised 68 nodes, corresponding to brain regions defined by the Desikan–Killiany atlas, and weighted edges derived from PLI values. For each frequency band (delta, theta, alpha, beta, gamma) and each epoch, we constructed an undirected weighted functional connectivity matrix of size 68 × 68. These matrices were then averaged across all epochs for each participant. To simplify the graph and reduce spurious connections, we applied binary thresholding. To reduce spurious connections, we binarized each connectivity matrix using a proportional threshold of κ = 0.20, retaining only the strongest 20% of connections; this approach is consistent with that of prior pediatric ASD studies (23, 26, 27). Because the chosen threshold can influence graph metrics, we also repeated all analyses using κ = 0.10, 0.12, …, 0.30 (10).

For the resulting binary matrices, we computed commonly used graph metrics: clustering coefficient (C), characteristic path length (L), and small-worldness (SW) (59). These metrics were computed for each frequency band and each κ value using the Brain Connectivity Toolbox (BCT, version 2019-03-03; https://sites.google.com/site/bctnet/). The mathematical definitions are detailed in previous literature (10, 60). The clustering coefficient (C) measures the tendency of nodes to form local clusters, reflecting the degree of segregated neural processing in the brain (10).

Mathematically, the characteristic path length L is defined as the average of the shortest path lengths between all pairs of nodes in a network, serving as an indicator of the efficiency of global information integration. However, readers should be aware of a subtle distinction between the mathematical definition and its implementation in the BCT. In each binary graph, we first computed the shortest-path distance matrix D according to the conventions of the BCT. After binarizing the adjacency matrix, we applied BCT’s function distance_bin of the BCT (https://github.com/fieldtrip/fieldtrip/blob/master/external/bct/distance_bin.m) so that each entry $D_{ij}$ represents the length of the shortest path between nodes i and j and is set to ∞ if no connecting path exists. We then invoked BCT’s function charpath(D, 0, 0) (https://github.com/jblocher/matlab-network-utilities/blob/master/BrainConnectivity/charpath.m), where the third argument infinite_dist = 0 instructs the function to (i) ignore all ∞ entries and (ii) compute the average over finite distances only (i.e., distances among nodes belonging to the same connected component). Specifically, charpath does not explicitly extract the largest connected component beforehand; rather, it treats every ∞ entry as missing and calculates


$$L=\frac{1}{\left|\{(i,j)|D_{ij}<\infty ,i<j\}\right|}\sum_{\begin{array}{c} i,j\\ D_{ij}<\infty ,i<j \end{array}}D_{ij}$$

Because our thresholded binary graphs typically yielded one large, connected component encompassing most nodes—and only a few isolated nodes or minor subcomponents appeared, particularly at lower proportional thresholds—ignoring ∞ values effectively approximates a restriction to the largest component in practice. Nonetheless, we emphasize that we did not manually remove small isolated components; instead, we relied on charpath(D,0,0) to drop only ∞ distances, thereby including all finite-distance pairs across all components (which differs subtly from the mathematical definition of L). We adopted this procedure because it aligns with prior MEG-based ASD studies (23, 26, 27) and with Brainstorm’s implementation of BCT measures, ensuring consistency and comparability with existing literature.

The small-worldness (SW) metric captures the balance between local specialization and global integration, a hallmark of efficient brain networks (60). It is defined as the ratio of the normalized clustering coefficient to the normalized characteristic path length, compared with equivalent random networks (61). To obtain the normalized metrics, we generated 1,000 random networks for each graph by rewiring all edges five times, preserving the same number of nodes and edges as the original network. We calculated the mean clustering coefficient (C_rand_) and the characteristic path length (L_rand_) for these random networks. The normalized metrics were computed as:


$$C_{norm}=\frac{C}{C_{rand}},L_{norm}=\frac{L}{L_{rand}}$$

The small-worldness ratio was calculated as


$$SW=\frac{C_{norm}}{L_{norm}}$$

For each participant, we obtained C, L, and SW values for each frequency band.

### 2.10 Statistical analyses

Statistical analyses were performed using Stata (version 17.0; StataCorp LLC, College Station, TX, USA). Group differences in age, K-ABC, and SRS scores between the ASD and TD participants were assessed using a two-tailed Student’s *t*-test. Sex differences were examined using the chi-square test. All categorical variables were expressed as numbers, while continuous parameters were expressed as means.

Our main objective was to investigate the effects of experimental conditions (DR vs. EO) on three graph measures: small-worldness (SW), clustering coefficient (C), and characteristic path length (L). Additionally, we examined whether EO-induced changes in graph measures correlated with autistic traits and whether these changes depended on baseline values from the DR condition.

To examine the effects of experimental conditions, we conducted separate linear mixed-effects regression analyses for each graph measure (SW, C, and L) across five frequency bands (delta, theta, alpha, beta, and gamma). Each model included fixed effects for diagnosis (ASD vs. TD), experimental conditions (DR vs. EO), their interaction, age, and sex, with a random intercept for each participant to account for within-subject correlations. This mixed effects approach allowed us to account for individual variability and the hierarchical structure of the data. Given that SW, C, and L quantify different aspects of the graph structure, but are not strictly independent, we applied a Bonferroni correction to adjust for multiple comparisons across the five frequency bands. Statistical significance was set at *p* < 0.01 (0.05/5) (62).

If significant diagnosis-by-experimental condition interaction was found in any model, we further investigated the relationship between autistic traits and changes in graph measures induced by the EO condition. We calculated the difference in each graph measure between the experimental conditions (EO minus DR) and performed linear regression analyses to predict the raw total SRS scores based on the EO-DR difference, diagnosis (ASD vs. TD), their interaction, and age and sex as covariates. Following Rubin (63), we treat these regressions as individual tests of distinct null hypotheses and set statistical significance at *p* < 0.05. Because the omnibus interaction served as a trigger, these analyses are presented as exploratory, hypothesis‐generating tests and should be interpreted with caution. For these follow-up subgroup analyses, while our primary inferential framework relies on a predefined threshold of α = 0.05 for determining statistical significance, we also report *p*-values between 0.05 and 0.10 as exploratory trends when they align with the broader pattern of results. Labeling a result as an exploratory trend does not imply confirmation, but rather highlights a potentially meaningful pattern that may merit replication. Importantly, all such trends are explicitly framed as exploratory rather than confirmatory. Readers are cautioned against overinterpreting these findings; true validation will require independent replication in larger samples.

To assess whether EO-induced changes depended on baseline measures obtained in the DR condition, we calculated the difference in each graph measure between conditions (EO minus DR) and used the baseline value (from the DR condition) to predict the difference. Separate linear regression analyses were conducted for each frequency band. The models included fixed effects for diagnosis (ASD vs. TD), baseline graph measures, their interaction terms, age, and sex. Again, we applied a Bonferroni correction to adjust for multiple comparisons across the five frequency bands. Statistical significance was set at *p <* 0.01 (0.05/5).

To ensure that key mixed-effects regression models met standard assumptions, we conducted a minimal set of diagnostic checks on the models central to our primary inferences. For each of these models, we visually inspected both residual and random-effects diagnostics. First, we assessed residual distributions by plotting histograms of raw residuals to verify approximate normality, ensuring the absence of pronounced skewness or kurtosis. Second, we evaluated homoscedasticity by plotting residuals against fitted values; the absence of a systematic funnel-like pattern suggested roughly constant variance across the range of predicted values. Third, we extracted estimated random intercepts for each participant and examined their distribution via histogram to confirm approximate normality (64, 65). For the linear regression models, we applied similar diagnostics; we plotted histograms of residuals to assess normality and scatterplots of residuals versus fitted values to evaluate homoscedasticity. As with the mixed-effects models, these checks were performed only for the primary models underpinning our main conclusions; diagnostic plots for other models are available upon request.

## 3 Results

### 3.1 Participants

Among the total of 39 children with ASD recruited for the study, 16 were excluded from the analyses: one boy with ASD had evident intellectual disability reflected in a K-ABC mental processing scale score <70; five boys and five girls exhibited excessive noise in their MEG recordings; and three boys and two girls were unable to complete their MRI recordings. Among the 62 children enrolled in the TD group, 31 children were excluded from the analysis: 13 boys and seven girls exhibited excessive noise in their MEG recordings, and five boys and six girls could not complete their MRI recordings.

Consequently, the final sample consisted of 23 children with ASD (14 boys and 9 girls) and 31 TD children (17 boys and 14 girls). The age range of children in the ASD group was 60–97 months, while that of the TD group was 44–109 months. There were no significant differences between groups in sex, age, number of available epochs, or K-ABC scale scores. However, total SRS scores were significantly different between the two groups (*t* = −6.879, *p* < 0.001). These findings are summarized in **Table 1**. For the ASD group, the mean scores of ADOS-2 were as follows: Social Affect score: 6.9 (standard deviation [SD] = 3.9), Restricted and Repetitive Behaviors score: 2.3 (SD = 1.5), total ADOS-2 score: 9.2 (SD = 4.6), and Comparison score: 5.1 (SD = 2.4).

**Table 1:** Participant characteristics.

Characteristic	ASD	TD	*χ^2^-* or *t-*values	*p*-value
N	23	31		
Sex ^†^	14	17	0.196	0.658
Month ^‡^	73.770	75.348	−0.480	0.634
Epoch number (DR) ^‡^	34.957	35.548	0.826	0.413
Epoch number (EO) ^‡^	21.391	22.193	0.716	0.477
K-ABC ^‡^	102.087	116.129	3.110	0.030
SRS ^‡^	69.391	47.677	−6.879	<0.001*

†Chi-square test; ‡ Student’s *t*-test; *Statistically significant; ASD, autism spectrum disorder; TD, typically developing children; DR, dark room; EO, eyes open; K-ABC, Kaufman Assessment Battery for Children; SRS, Social Responsiveness Scale.

### 3.2 Effect of experimental condition on graph measures

Separate linear mixed-effects regression analyses were conducted for each graph measure (SW, C, and L) across all frequency bands (delta, theta, alpha, beta, and gamma). The models included fixed effects for diagnosis (ASD vs. TD), experimental condition (DR vs. EO), their interaction term, age, and sex, with a random intercept for each participant to account for within-subject correlations. Detailed results from these analyses are presented in **Table 2**.

**Table 2:** Effects of diagnosis, experimental condition, and their interaction on graph metrics.

Graph measures	Frequency band	Predictor	Coefficient	S.E.	*z*-value	*p*-value	95% CI		
SW	Delta	Diagnosis (ASD vs. TD)	−0.038	0.022	−1.733	0.083	−0.080	–	0.005
		Experimental condition (DR vs. EO)	−0.035	0.019	−1.875	0.061	−0.072	–	0.002
		Diagnosis * Experimental condition	0.075	0.029	2.579	0.010*	0.018	–	0.131
		Age	−0.001	0.001	−1.123	0.261	−0.002	–	0.001
		Sex	0.018	0.016	1.132	0.258	−0.014	–	0.050
	Theta	Diagnosis (ASD vs. TD)	−0.012	0.020	−0.607	0.544	−0.051	–	0.027
		Experimental condition (DR vs. EO)	−0.032	0.018	−1.771	0.077	−0.068	–	0.003
		Diagnosis × Experimental condition	0.012	0.028	0.413	0.679	−0.043	–	0.066
		Age	−0.001	0.001	−1.404	0.160	−0.002	–	0.000
		Sex	−0.004	0.014	−0.280	0.779	−0.032	–	0.024
	Alpha	Diagnosis (ASD vs. TD)	0.006	0.021	0.286	0.775	−0.036	–	0.048
		Experimental condition (DR vs. EO)	0.031	0.019	1.626	0.104	−0.006	–	0.068
		Diagnosis × Experimental condition	0.008	0.029	0.270	0.787	−0.049	–	0.065
		Age	0.000	0.001	0.761	0.447	−0.001	–	0.002
		Sex	0.031	0.016	2.010	0.044	0.001	–	0.062
	Beta	Diagnosis (ASD vs. TD)	−0.062	0.021	−2.992	0.003	−0.102	–	−0.021
		Experimental condition (DR vs. EO)	−0.026	0.018	−1.430	0.153	−0.061	–	0.010
		Diagnosis × Experimental condition	0.079	0.028	2.843	0.004*	0.024	–	0.133
		Age	0.001	0.001	1.874	0.061	0.000	–	0.002
		Sex	0.023	0.015	1.487	0.137	−0.007	–	0.053
	Gamma	Diagnosis (ASD vs. TD)	−0.010	0.022	−0.468	0.639	−0.054	–	0.033
		Experimental condition (DR vs. EO)	0.003	0.020	0.149	0.881	−0.036	–	0.042
		Diagnosis × Experimental condition	−0.003	0.031	−0.083	0.933	−0.063	–	0.058
		Age	0.001	0.001	1.497	0.134	0.000	–	0.002
		Sex	0.009	0.016	0.574	0.566	−0.022	–	0.041
C	Delta	Diagnosis (ASD vs. TD)	−0.009	0.007	−1.250	0.211	−0.023	–	0.005
		Experimental condition (DR vs. EO)	0.000	0.006	−0.038	0.969	−0.011	–	0.011
		Diagnosis × Experimental condition	0.016	0.009	1.812	0.070	−0.001	–	0.033
		Age	0.000	0.000	−0.339	0.734	−0.001	–	0.000
		Sex	0.006	0.006	1.018	0.309	−0.005	–	0.017
	Theta	Diagnosis (ASD vs. TD)	0.002	0.010	0.218	0.828	−0.017	–	0.021
		Experimental condition (DR vs. EO)	0.017	0.009	1.852	0.064	−0.001	–	0.034
		Diagnosis × Experimental condition	−0.019	0.014	−1.398	0.162	−0.046	–	0.008
		Age	−0.001	0.000	−1.880	0.060	−0.001	–	0.000
		Sex	−0.005	0.007	−0.707	0.480	−0.018	–	0.009
	Alpha	Diagnosis (ASD vs. TD)	−0.011	0.019	−0.555	0.579	−0.048	–	0.027
		Experimental condition (DR vs. EO)	0.046	0.018	2.625	0.009	0.012	–	0.080
		Diagnosis × Experimental condition	−0.033	0.027	−1.217	0.224	−0.085	–	0.020
		Age	−0.001	0.001	−1.433	0.152	−0.002	–	0.000
		Sex	−0.008	0.014	−0.559	0.576	−0.035	–	0.019
	Beta	Diagnosis (ASD vs. TD)	−0.012	0.011	−1.033	0.302	−0.034	–	0.010
		Experimental condition (DR vs. EO)	0.001	0.009	0.056	0.955	−0.018	–	0.019
		Diagnosis × Experimental condition	0.001	0.014	0.072	0.942	−0.027	–	0.029
		Age	−0.001	0.000	−2.198	0.028	−0.002	–	0.000
		Sex	0.008	0.009	0.938	0.348	−0.009	–	0.025
	Gamma	Diagnosis (ASD vs. TD)	0.007	0.013	0.512	0.609	−0.019	–	0.032
		Experimental condition (DR vs. EO)	0.016	0.008	2.006	0.045	0.000	–	0.031
		Diagnosis × Experimental condition	−0.008	0.012	−0.658	0.510	−0.032	–	0.016
		Age	−0.001	0.000	−1.091	0.275	−0.001	–	0.000
		Sex	0.007	0.012	0.576	0.564	−0.016	–	0.029
L	Delta	Diagnosis (ASD vs. TD)	0.006	0.008	0.705	0.481	−0.010	–	0.021
		Experimental condition (DR vs. EO)	0.010	0.007	1.400	0.161	−0.004	–	0.025
		Diagnosis × Experimental condition	−0.011	0.011	−0.955	0.340	−0.033	–	0.011
		Age	0.000	0.000	1.478	0.139	0.000	–	0.001
		Sex	0.000	0.006	−0.051	0.959	−0.011	–	0.011
	Theta	Diagnosis (ASD vs. TD)	0.005	0.009	0.521	0.602	−0.013	–	0.023
		Experimental condition (DR vs. EO)	0.017	0.008	1.976	0.048	0.000	–	0.033
		Diagnosis × Experimental condition	−0.020	0.013	−1.552	0.121	−0.046	–	0.005
		Age	0.000	0.000	0.354	0.723	0.000	–	0.001
		Sex	−0.003	0.007	−0.469	0.639	−0.016	–	0.010
	Alpha	Diagnosis (ASD vs. TD)	−0.022	0.015	−1.501	0.133	−0.051	–	0.007
		Experimental condition (DR vs. EO)	−0.016	0.012	−1.299	0.194	−0.040	–	0.008
		Diagnosis × Experimental condition	−0.011	0.019	−0.557	0.578	−0.048	–	0.026
		Age	0.000	0.000	0.464	0.642	−0.001	–	0.001
		Sex	−0.020	0.011	−1.780	0.075	−0.042	–	0.002
	Beta	Diagnosis (ASD vs. TD)	−0.002	0.012	−0.129	0.898	−0.025	–	0.022
		Experimental condition (DR vs. EO)	0.003	0.011	0.290	0.772	−0.018	–	0.024
		Diagnosis × Experimental condition	−0.006	0.017	−0.392	0.695	−0.039	–	0.026
		Age	−0.001	0.000	−1.734	0.083	−0.001	–	0.000
		Sex	0.005	0.008	0.543	0.587	−0.012	–	0.021
	Gamma	Diagnosis (ASD vs. TD)	0.010	0.011	0.869	0.385	−0.012	–	0.032
		Experimental condition (DR vs. EO)	0.016	0.010	1.535	0.125	−0.004	–	0.036
		Diagnosis × Experimental condition	−0.018	0.016	−1.174	0.240	−0.049	–	0.012
		Age	−0.001	0.000	−2.112	0.035	−0.001	–	0.000
		Sex	0.006	0.008	0.785	0.432	−0.010	–	0.023
SW	Delta	**TD**							
		Experimental condition (DR vs. EO)	−0.035	0.019	−1.906	0.057	−0.072	–	0.001
		Age	−0.001	0.001	−1.520	0.129	−0.003	–	0.000
		Sex	−0.002	0.019	−0.109	0.913	−0.039	–	0.035
		**ASD**							
		Experimental condition (DR vs. EO)	0.039	0.022	1.777	0.076	−0.004	–	0.082
		Age	0.000	0.001	−0.288	0.773	−0.003	–	0.002
		Sex	0.045	0.028	1.592	0.111	−0.010	–	0.100
	Beta	**TD**							
		Experimental condition (DR vs. EO)	−0.026	0.015	−1.713	0.087	−0.055	–	0.004
		Age	0.001	0.001	1.466	0.143	0.000	–	0.003
		Sex	0.014	0.021	0.660	0.509	−0.028	–	0.056
		**ASD**							
		Experimental condition (DR vs. EO)	0.053	0.023	2.293	0.022*	0.008	–	0.098
		Age	0.001	0.001	0.913	0.361	−0.001	–	0.003
		Sex	0.035	0.024	1.493	0.135	−0.011	–	0.082

*Statistically significant; ASD, autism spectrum disorder; TD, typically developing children; SW, small-worldness; C, clustering coefficient; L, characteristic path length; DR, dark room; EO, eyes open; CI, confidence interval; S.E., standard error.

#### 3.2.3 Clustering coefficient (C)

**Figure 1:**
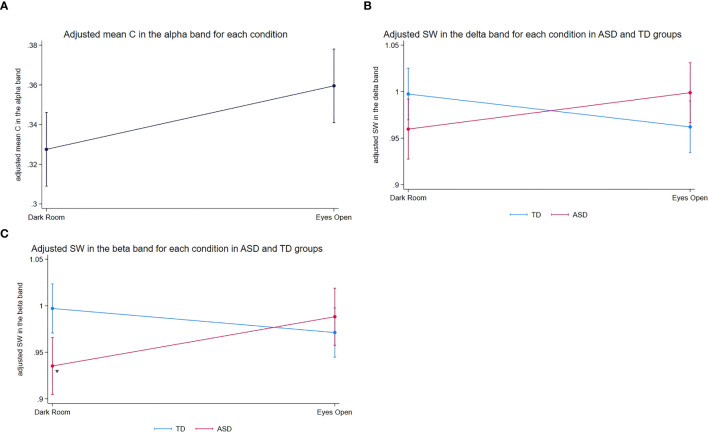
Adjusted mean graph measures across experimental conditions in ASD and TD groups. **(A)** Adjusted mean *C* in the alpha frequency band for each experimental condition (DR vs. EO), adjusted for age and sex. The means were estimated from a linear mixed-effects model, including fixed effects for experimental condition, diagnosis, their interaction, age, and sex, with a random intercept for each participant to account for within-subject correlations. Since the diagnosis-by-condition interaction for C was not significant, only the main effect of the experimental conditions is presented. Error bars represent the standard error of the adjusted means. *C* was higher in the EO condition than that in the DR condition. **(B)** Adjusted mean SW in the delta frequency band for each experimental condition (DR vs. EO) in the ASD and TD groups. Error bars represent the standard error of the mean adjusted for age and sex. The figure illustrates the diagnosis-by-experimental condition interaction, with the ASD group showing a trend toward increased SW and the TD group showing a trend toward decreased SW from DR to EO condition. **(C)** Adjusted mean SW in the beta frequency band for each experimental condition (DR vs. EO) in the ASD and TD groups. Error bars represent the standard error of the mean, adjusted for age and sex. An asterisk (*) denotes a significant increase in SW for the ASD group from DR. ASD, autism spectrum disorder; C, clustering coefficient; EO, eyes open; DR, dark room; SW, small-worldness; TD, typically developing.

In the alpha frequency band, we found a significant main effect of the experimental condition on C (*z* = 2.62, *p* = 0.0087), indicating that C was higher in the EO condition compared to that in the DR condition across both groups (**Figure 1A**).

#### 3.2.4 Small-worldness

In the delta frequency band, we observed a significant diagnosis‐by‐experimental‐condition interaction on SW (*z* = 2.58, *p* = 0.0099). In follow-up exploratory, hypothesis-generating analyses, children with ASD showed a trend toward increased SW from DR to EO (*z* = 1.78, *p* = 0.0756), whereas TD children displayed a trend in the opposite direction (*z* = −1.91, *p* = 0.0567). Neither effect reached the conventional threshold of α = 0.05, and both are therefore reported as exploratory trends to be interpreted with caution. Altogether, these results indicate that the EO condition may modulate delta‐band SW differently in children with ASD than in TD children (**Figure 1B**).

In the beta frequency band, we found a significant diagnosis‐by‐experimental‐condition interaction on SW (*z* = 2.84, *p* = 0.0045), as well as a significant main effect of diagnosis (*z* = −2.99, *p* = 0.0028). Group-specific analyses revealed that SW significantly increased from DR to EO in children with ASD (*z* = 2.29, *p* = 0.0219), whereas TD children exhibited a trend toward decreased SW (*z* = −1.71, *p* = 0.0867). As the TD effect did not reach significance, it is also reported as an exploratory trend. Together, these results suggest that the EO condition modulates beta‐band SW differently in TD children and children with ASD (**Figure 1C**).

Diagnostic plots for the abovementioned mixed-effects models are provided in **Supplementary Figures 1, 2**. These checks revealed qualitatively similar patterns across models. The residuals were symmetrically distributed and clustered near zero, with no clear signs of heteroscedasticity, and the random intercepts approximated a Gaussian distribution, thereby supporting the validity of our inferential framework.

### 3.2.5 Relationship between changes induced by the EO condition and autistic traits

Given the significant diagnosis-by-experimental condition interactions observed in the models predicting SW in the delta and beta frequency bands, we further examined the relationship between autistic traits and EO-induced changes in SW. To do this, we calculated the difference in SW (ΔSW) between the EO and DR conditions (EO minus DR) for each participant. We then performed linear regression analyses to predict raw total SRS scores based on this difference, including diagnosis, their interaction term, age, and sex as predictors. Some of these regression models did not satisfy the assumption of homoscedasticity, necessitating the application of heteroscedasticity-robust standard errors (66).

#### 3.2.6 Delta frequency band

**Figure 2:**
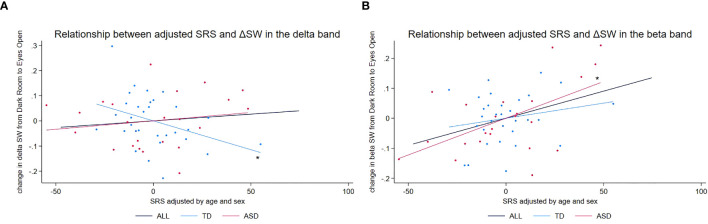
Scatter plots of adjusted SRS scores vs. adjusted change in SW in beta and delta frequency bands. **(A)** Scatter plot of adjusted SRS scores vs. adjusted change in SW (ΔSW) in the delta frequency band. An asterisk (*) denotes a significant negative relationship between adjusted ΔSW and adjusted SRS scores (*t* = −2.475, *p* = 0.019), suggesting that lower SRS scores (milder autistic traits) in TD children are associated with larger increases in SW from the DR to the EO condition. **(B)** Scatter plot of adjusted SRS scores vs. adjusted change in SW (ΔSW) in the beta frequency band. An asterisk (*) denotes a significant positive relationship between adjusted ΔSW and adjusted SRS scores (*t* = 2.995, *p* = 0.007), suggesting that higher SRS scores (more pronounced autistic traits) in children with ASD are associated with larger increases in SW from the DR to the EO condition. ASD, autism spectrum disorder; DR, dark room; EO, eyes open; SRS, Social Responsiveness Scale; SW, small-worldness; TD, typically developing children.

In the delta band (**Figure 2A**), there was a significant main effect of diagnosis on SRS scores (*t* = 7.27, *p* < 0.001), reflecting the differences in SRS scores between the ASD and TD groups. We also observed an exploratory trend for the interaction diagnosis × ΔSW (*t =* 2.00, *p* = 0.051). In follow-up, hypothesis-generating, group-specific analyses—reported as exploratory due to *p*-values exceeding the conventional threshold α = 0.05—we found for the ASD group no significant association between ΔSW and SRS (*t =* 0.93, *p* = 0.365) and for the TD group a significant negative association (*t =* −2.61, *p* = 0.014), indicating that larger SW increases from DR to EO were linked to lower SRS scores (i.e., milder autistic traits).

#### 3.2.6 Beta frequency band

In the beta frequency band (**Figure 2B**), we found a significant main effect of diagnosis (*t* = 4.92, *p* < 0.001). An exploratory trend for the interaction diagnosis × ΔSW also emerged (*t =* 1.79, *p* = 0.080). In hypothesis-generating, group-specific analyses—again treated as exploratory given the nonsignificant interaction—we found for the ASD group a significant positive association (*t =* 2.67, *p* = 0.015), such that larger SW increases from DR to EO corresponded with higher SRS scores (i.e., more pronounced autistic traits), whereas no significant association was observed for the TD group (*t =* 1.08, *p* = 0.289).

Because the overall interactions were only exploratory trends (i.e., they exceeded the conventional threshold α = 0.05), these subgroup results should be interpreted cautiously and regarded as hypothesis-generating rather than confirmatory. Replication in a larger sample is needed to evaluate the robustness of these patterns.

Detailed results from these analyses are presented in **Table 3** and **Figure 2**. Diagnostic plots for the corresponding linear regression models are provided in **Supplementary Figure 3**.

**Table 3:** Relationship between changes induced by the EO condition and autistic traits.

vs. raw total SRS scores		Coefficient	S.E.	*t*-value	*p*-value	95% CI		
SW in the delta band	EO-induced changes in SW (EO − DR)	−67.257	25.941	−2.593	0.013	−119.416	–	−15.099
	Diagnosis	42.120	5.791	7.273	<0.001*	30.476	–	53.765
	Diagnosis × EO-induced changes in SW	106.735	53.361	2.000	0.051	−0.554	–	214.024
	Age	0.312	0.259	1.205	0.234	−0.209	–	0.833
	Sex	9.736	6.122	1.590	0.118	−2.574	–	22.046
(Group-specific analysis)	TD							
	EO-induced changes in SW (EO − DR)	−60.015	22.969	−2.613	0.014	−107.144	–	−12.886
	Age	−0.054	0.183	−0.296	0.769	−0.429	–	0.320
	Sex	11.676	5.962	1.958	0.061	−0.557	–	23.909
	ASD							
	EO-induced changes in SW (EO − DR)	42.794	46.134	0.928	0.365	−53.765	–	139.352
	Age	1.061	0.503	2.111	0.048	0.009	–	2.113
	Sex	3.797	11.915	0.319	0.753	−21.141	–	28.735
SW in the beta band	EO-induced changes in SW (EO − DR)	29.549	37.499	0.788	0.435	−45.847	–	104.945
	Diagnosis	33.803	6.873	4.918	<0.001*	19.984	–	47.623
	Diagnosis × EO-induced changes in SW	107.043	59.820	1.789	0.080	−13.233	–	227.320
	Age	0.089	0.242	0.368	0.715	−0.398	–	0.576
	Sex	7.088	5.953	1.191	0.240	−4.882	–	19.057
(Group-specific analysis)	TD							
	EO-induced changes in SW (EO − DR)	41.222	38.089	1.082	0.289	−36.929	–	119.373
	Age	−0.217	0.179	−1.211	0.236	−0.586	–	0.151
	Sex	10.282	5.827	1.764	0.089	−1.675	–	22.238
	ASD							
	EO-induced changes in SW (EO − DR)	128.138	47.905	2.675	0.015	27.872	–	228.404
	Age	0.744	0.433	1.720	0.102	−0.161	–	1.650
	Sex	0.597	11.049	0.054	0.957	−22.528	–	23.722

*Statistically significant; ASD, autism spectrum disorder; TD, typically developing children; SW, small-worldness; SRS, Social Responsiveness Scale; S.E., standard error; CI, confidence interval; DR, dark room; EO, eyes open.

To visualize the results from the linear regression models, we adjusted SRS raw scores and changes in SW (ΔSW) for age and sex using the following steps: (1) we regressed SRS scores on age and sex to obtain the residuals (adjusted SRS scores) for each group; (2) we regressed ΔSW on age and sex to obtain residuals (adjusted ΔSW) for each group; and (3) we generated scatter plots of adjusted SRS scores versus adjusted ΔSW for each group, including regression lines to illustrate the relationships.

### 3.3 Relationship between EO-induced changes and baseline measures

Given the established differences in resting-state graph measures between ASD and TD populations (23, 24, 29–31), we investigated whether EO-induced changes depended on baseline measures obtained in the DR condition. To assess this we: Calculated the difference in each graph measure between the conditions (EO minus DR); used the baseline value (DR condition) to predict this difference; and conducted separate linear regression analyses for each frequency band, including fixed effects for diagnosis (ASD vs. TD), baseline graph measures, interaction terms (diagnosis × baseline measures), age, and sex.

#### 3.3.1 Clustering coefficient (C)

**Figure 3:**
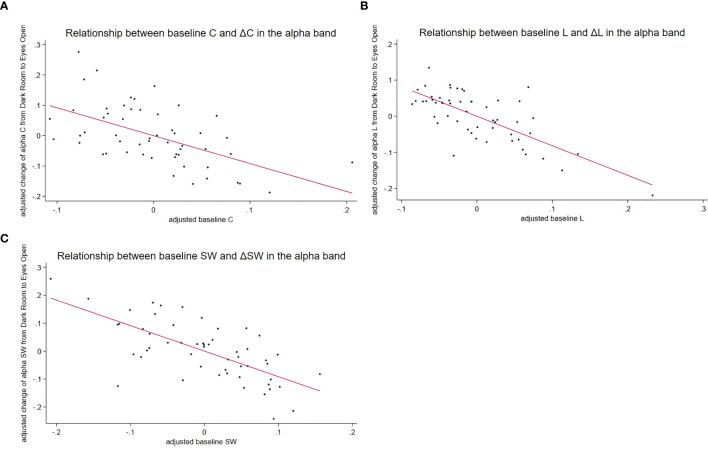
Relationship between baseline measures and changes induced by the EO condition in the alpha frequency band. This figure illustrates the negative correlations between baseline graph measures obtained in the DR condition and changes induced by the EO condition, with the strongest relationship observed in the alpha frequency band. Baseline measures and changes were adjusted for age and sex using residuals from regression models. Each data point represents an individual participant from the ASD and TD groups. Negative correlations across all three graph metrics suggest that baseline network properties influence the extent of change induced by visual input in the alpha frequency band. **(A)** Scatter plot of the relationship between the adjusted baseline *C* and the adjusted change in *C* (EO minus DR). The downward slope indicated that participants with higher baseline *C* exhibited smaller increases in *C* when transitioning from the DR to the EO condition. The solid line represents the linear regression fit. **(B)** Scatter plot of the relationship between the adjusted baseline L and the adjusted change in L. The downward slope indicated that participants with a higher baseline L exhibited smaller increases in L when transitioning from DR to EO. The solid line represents the linear regression fit. **(C)** Scatter plot of the relationship between the adjusted baseline SW and the adjusted change in SW. The downward slope indicated that participants with higher baseline SW values exhibited smaller increases in SW when moving from the DR to the EO condition. The solid line represents the linear regression fit. baseline *C*, clustering coefficient under the DR condition; baseline L, characteristic path length in the DR condition; baseline SW, small-worldness at DR condition; ASD, autism spectrum disorder; TD, typically developing children; DR, dark room; EO, eyes open.

Significant effects of the baseline *C* (**Figure 3A**) were observed in the delta (*t* = −4.59, *p* < 0.001), theta (*t* = −4.09, *p* < 0.001), and alpha (*t* = −3.35, *p* = 0.002) frequency bands. The key findings were as follows: No significant diagnosis-by-baseline interactions were found, no significant effects of age or sex were observed, and a higher baseline C was associated with a smaller increase in *C* from DR to EO in these frequency bands. The findings are summarized in **Table 4**. Diagnostic plots for the corresponding linear regression models are provided in **Supplementary Figure 4**.

**Table 4:** Effects of baseline graph measures on changes induced by the EO condition.

Graph measures	Frequency band	Predictor	Coefficient	S.E.	*t*-value	*p*-value	95% CI		
SW	Delta	Baseline measure (DR)	−0.986	0.209	−4.719	<0.001*	−1.406	–	−0.566
		Diagnosis	−0.154	0.273	−0.567	0.574	−0.703	–	0.394
		Diagnosis × Baseline measure (DR)	0.198	0.277	0.714	0.479	−0.360	–	0.756
		Age	0.000	0.001	−0.144	0.886	−0.002	–	0.002
		Sex	0.031	0.022	1.366	0.178	−0.014	–	0.076
	Theta	Baseline measure (DR)	−1.141	0.159	−7.175	<0.001*	−1.461	–	−0.821
		Diagnosis	−0.243	0.230	−1.055	0.297	−0.706	–	0.220
		Diagnosis × Baseline measure (DR)	0.242	0.232	1.042	0.302	−0.225	–	0.710
		Age	0.000	0.001	−0.001	0.999	−0.002	–	0.002
		Sex	0.016	0.019	0.835	0.408	−0.022	–	0.053
	Alpha	Baseline measure (DR)	−0.920	0.181	−5.091	<0.001*	−1.283	–	−0.556
		Diagnosis	0.006	0.273	0.021	0.983	−0.544	–	0.556
		Diagnosis × Baseline measure (DR)	0.010	0.290	0.033	0.974	−0.573	–	0.592
		Age	0.000	0.001	0.394	0.695	−0.002	–	0.002
		Sex	0.007	0.024	0.288	0.775	−0.041	–	0.054
	Beta	Baseline measure (DR)	−0.659	0.191	−3.459	0.001	−1.043	–	−0.276
		Diagnosis	0.446	0.261	1.711	0.093	−0.078	–	0.970
		Diagnosis × Baseline measure (DR)	−0.439	0.269	−1.633	0.109	−0.979	–	0.102
		Age	0.002	0.001	2.422	0.019	0.000	–	0.004
		Sex	0.029	0.021	1.421	0.162	−0.012	–	0.071
	Gamma	Baseline measure (DR)	−0.927	0.231	−4.018	<0.001*	−1.390	–	−0.463
		Diagnosis	−0.017	0.316	−0.053	0.958	−0.652	–	0.619
		Diagnosis × Baseline measure (DR)	0.005	0.327	0.016	0.988	−0.652	–	0.662
		Age	0.002	0.001	1.503	0.139	−0.001	–	0.004
		Sex	−0.013	0.025	−0.500	0.620	−0.064	–	0.038
C	Delta	Baseline measure (DR)	−0.906	0.197	−4.590	<0.001*	−1.303	–	−0.509
		Diagnosis	−0.148	0.095	−1.550	0.128	−0.340	–	0.044
		Diagnosis × Baseline measure (DR)	0.625	0.379	1.651	0.105	−0.136	–	1.386
		Age	0.000	0.000	−0.264	0.793	−0.001	–	0.001
		Sex	0.006	0.008	0.774	0.443	−0.010	–	0.022
	Theta	Baseline measure (DR)	−1.001	0.245	−4.089	<0.001*	−1.493	–	−0.509
		Diagnosis	0.014	0.095	0.148	0.883	−0.177	–	0.205
		Diagnosis × Baseline measure (DR)	−0.114	0.362	−0.315	0.754	−0.842	–	0.614
		Age	−0.001	0.000	−2.523	0.015	−0.002	–	0.000
		Sex	−0.009	0.011	−0.795	0.431	−0.031	–	0.013
	Alpha	Baseline measure (DR)	−0.832	0.248	−3.352	0.002	−1.330	–	−0.333
		Diagnosis	0.050	0.129	0.387	0.700	−0.209	–	0.308
		Diagnosis × Baseline measure (DR)	−0.284	0.390	−0.728	0.470	−1.067	–	0.500
		Age	−0.001	0.001	−1.543	0.129	−0.003	–	0.000
		Sex	0.008	0.023	0.328	0.744	−0.039	–	0.055
	Beta	Baseline measure (DR)	−0.560	0.215	−2.605	0.012	−0.993	–	−0.128
		Diagnosis	0.078	0.087	0.901	0.372	−0.097	–	0.254
		Diagnosis × Baseline measure (DR)	−0.321	0.316	−1.016	0.315	−0.955	–	0.314
		Age	0.000	0.001	−0.553	0.583	−0.001	–	0.001
		Sex	0.030	0.012	2.397	0.020	0.005	–	0.054
	Gamma	Baseline measure (DR)	−0.236	0.177	−1.332	0.189	−0.592	–	0.120
		Diagnosis	0.036	0.076	0.476	0.636	−0.117	–	0.190
		Diagnosis × Baseline measure (DR)	−0.164	0.282	−0.581	0.564	−0.730	–	0.403
		Age	0.000	0.001	0.546	0.588	−0.001	–	0.001
		Sex	0.013	0.012	1.056	0.296	−0.012	–	0.038
L	Delta	Baseline measure (DR)	−1.521	0.282	−5.396	<0.001*	−2.088	–	−0.954
		Diagnosis	−1.413	0.744	−1.898	0.064	−2.909	–	0.084
		Diagnosis × Baseline measure (DR)	0.740	0.391	1.893	0.064	−0.046	–	1.525
		Age	0.000	0.000	0.296	0.769	−0.001	–	0.001
		Sex	−0.004	0.010	−0.393	0.696	−0.023	–	0.016
	Theta	Baseline measure (DR)	−1.157	0.176	−6.570	<0.001*	−1.511	–	−0.803
		Diagnosis	−0.551	0.474	−1.163	0.251	−1.505	–	0.402
		Diagnosis × Baseline measure (DR)	0.282	0.249	1.133	0.263	−0.218	–	0.782
		Age	0.000	0.000	−1.232	0.224	−0.001	–	0.000
		Sex	−0.012	0.009	−1.352	0.183	−0.031	–	0.006
	Alpha	Baseline measure (DR)	−0.750	0.156	−4.804	<0.001*	−1.064	–	−0.436
		Diagnosis	0.395	0.444	0.890	0.378	−0.498	–	1.289
		Diagnosis × Baseline measure (DR)	−0.217	0.227	−0.956	0.344	−0.674	–	0.240
		Age	0.000	0.001	−0.022	0.982	−0.001	–	0.001
		Sex	−0.016	0.014	−1.122	0.268	−0.045	–	0.013
	Beta	Baseline measure (DR)	−1.053	0.238	−4.421	<0.001*	−1.532	–	−0.574
		Diagnosis	−0.144	0.620	−0.233	0.817	−1.391	–	1.103
		Diagnosis × Baseline measure (DR)	0.070	0.323	0.218	0.829	−0.579	–	0.719
		Age	−0.001	0.001	−0.959	0.342	−0.002	–	0.001
		Sex	0.024	0.013	1.820	0.075	−0.003	–	0.051
	Gamma	Baseline measure (DR)	−1.020	0.269	−3.791	<0.001*	−1.561	–	−0.479
		Diagnosis	−0.615	0.868	−0.708	0.482	−2.359	–	1.130
		Diagnosis × Baseline measure (DR)	0.316	0.454	0.697	0.489	−0.596	–	1.228
		Age	−0.001	0.001	−1.219	0.229	−0.002	–	0.000
		Sex	0.014	0.014	0.961	0.341	−0.015	–	0.042

*Statistically significant; SW, small-worldness; C, clustering coefficient; L, characteristic path length; S.E., standard error; CI, confidence interval; DR, dark room.

#### 3.3.2 Characteristic path length (L)

Baseline measures significantly predicted changes in L (**Figure 3B**) across all frequency bands (delta: *t* = −5.40, *p* < 0.001; theta: *t* = −6.57, *p* < 0.001; alpha: *t* = −4.80, *p* < 0.001, beta: *t* = −4.42, *p* = 0.0001; and gamma: *t* = −3.79, *p* = 0.004). No significant interactions or effects of age and sex were observed. This indicates that higher baseline L corresponds to smaller increases in L from DR to EO. The details are provided in **Table 4**.

#### 3.3.3 Small-worldness

Baseline SW (**Figure 3C**) significantly predicted changes in SW across all frequency bands: (delta: *t* = −4.72, *p* < 0.001; theta: *t* = −7.17, *p* < 0.001; alpha: *t* = −5.09, *p* < 0.001; beta: *t* = −3.46, *p* = 0.001; gamma: *t* = −4.02, *p* = 0.002). Again, no significant interactions or effects of age or sex were observed. A higher baseline SW corresponded to a smaller increase from the DR to the EO. (**Table 4**, **Figure 3**).

### 3.3.4 Threshold variability

To ensure that the binary‐graph results were not driven by an arbitrary choice of network density, we recomputed C, L, and SW across a range of proportional thresholds from 10% to 30% in 2% increments. As shown in **Supplementary Tables 1-3**, **Document 1**, the overall pattern of our findings remained qualitatively consistent across this density range, although *z*‐scores varied slightly at the extremes (below ~12% or above ~28% density). In other words, while absolute metric values changed with the chosen threshold, the key group and condition effects remained stable over a reasonable window of network densities.

## 4 Discussion

In this study, we examined how transitioning from DR to EO conditions affects three key graph-theoretical measures—small-worldness (SW), clustering coefficient (*C*), and characteristic path length (L)—across five frequency bands (delta, theta, alpha, beta, and gamma) in children with and without ASD. Our results revealed several important patterns: First, we observed the main effects of the experimental conditions on the clustering coefficient in the alpha band, indicating an overall higher clustering coefficient in the EO condition compared to the DR condition for both groups. Second, we examined whether these EO-induced changes depended on baseline (DR) measures, revealing a consistent pattern across all frequency bands: higher baseline values for clustering coefficients, characteristic path length, and SW corresponded to smaller increases in EO. Third, we identified significant diagnosis-by-condition interactions for SW in the delta and beta bands. While children with ASD tended to show increases in SW from DR to EO, TD children exhibited the opposite trend, suggesting that functional network reactivity to visual input differs between groups. Fourth, we found that the magnitude of the SW change in the beta band was positively associated with autistic trait severity in the ASD group (larger increases from DR to EO were linked to higher SRS scores). In contrast, SW changes in the delta band were negatively correlated with trait severity in the TD group (larger increases were linked to lower SRS scores). These findings suggest that functional network adaptation to visual input differs between ASD and TD children and that autistic trait severity is associated with specific patterns of SW modulation in response to environmental changes.

To the best of our knowledge, this is the first study to demonstrate differences in key graph-theoretical metrics between DR and EO conditions in children with and without ASD. Notably, we found that the clustering coefficient in the alpha band was higher in the EO condition than that in the DR condition. Since DR is considered analogous to EC conditions (44), our findings contrast with studies in adults that consistently report higher alpha-band clustering coefficients in EC relative to EO in both young and older adults (17–19, 67). This discrepancy suggests an age-dependent shift in functional network organization. Thus, our study extends the existing evidence by showing that while adults transition toward more global processing under EO, children’s functional networks shift toward more localized processing in response to visual input. Moreover, our results indicate that higher baseline values of the clustering coefficient, characteristic path length, and small-worldness correspond to smaller EO-induced increases. One possible interpretation is that, if a functional brain network is already optimized for visual processing, additional sensory input may induce only minimal changes. This perspective aligns with our findings when considering the study by Kavčič et al. (68), which documented an age-related increase in alpha-band clustering coefficients under EC in individuals aged 5–18 years. Although no study has directly compared baseline (EC) clustering coefficients between children and adults, it is plausible—given Kavčič et al.’s results—that our pediatric participants (with or without ASD) had lower baseline clustering coefficients than the adults in earlier studies (17–19, 67). Such age-related differences could explain why the clustering coefficients in children increase more under EO than under EC, whereas adults show the opposite pattern; namely, a higher baseline clustering coefficient in adults leads to a smaller or even negative change under EO. Together, these observations highlight an age-dependent mechanism of network reactivity in the alpha band, underscoring the need to further investigate how diagnostic status (ASD vs. TD) modulates this reactivity.

We identified significant diagnosis-by-condition interactions for SW in the delta and beta bands. Children with ASD tended to show increases in SW when transitioning from DR to EO, whereas TD children exhibited the opposite trend. A supplementary *t*-test indicated that TD children had significantly higher SW in the beta band (t(52) = 2.76, *p* = 0.008) and a trend toward higher SW in the delta band (t(52) = 1.66, *p* = 0.10). Given our earlier finding that higher baseline SW corresponds to smaller increases from DR to EO, these results suggest that elevated baseline SW in children with TD may partly explain the smaller (or even more negative) changes in SW during EO. In contrast, lower baseline SW in children with ASD may predispose them to greater increases in SW during EO. Although no previous studies have directly examined how EO vs. DR (EC) conditions affect graph measures in children with ASD, our data reveal distinct diagnostic differences in network reactivity. A crucial next step is to explore whether these differences in SW reactivity are associated with autistic trait severity. To address this, we examined how SW changes from DR to EO correlate with clinical measures of autistic traits, providing insight into the potential relationship between network reactivity and the core features of ASD.

Our findings reveal a previously unreported link between changes in SW and autistic traits. In children with ASD, larger increases in beta-band SW when transitioning from DR to EO were associated with more pronounced autistic traits, whereas in TD children, greater increases in delta-band SW were linked to less pronounced autistic traits. Although no prior studies have directly investigated these relationships, several hypotheses may help to interpret the observed patterns. First, beta band activity has been implicated in processes such as sensory integration (69, 70) and attention (71), both of which can be atypical in ASD. Given that higher SW reflects a more optimized functional network, this heightened reactivity in children with ASD who exhibit more pronounced autistic traits might represent a compensatory response to a less optimal network organization in the beta band when faced with visual processing demands. Second, while delta-band activity has traditionally been associated with long-term memory formation (72), recent studies indicate that delta-band oscillations also play a critical role in speech processing and comprehension (73–75)—functions closely tied to human communication. Consequently, delta-band changes in TD children may reflect more flexible network adaptations. Those who exhibit stronger shifts toward higher SW in this low-frequency range may show better speech comprehension, which, in turn, could be linked to fewer autistic-like traits. However, it is essential to recognize that the SRS may not capture identical underlying neurophysiological processes in TD children vs. those with ASD. In TD children, SRS scores could reflect cognitive functions relevant to communication, such as speech processing, whereas in children with ASD, these scores may indicate core etiological features of the condition, including excitatory/inhibitory imbalances (76).

Notably, our study findings were only observed in specific frequency bands. Eye‐state‐dependent changes in clustering coefficients emerged primarily in the alpha band—which is known to index attentional control (77–80)—across both groups. In contrast, diagnosis-by-condition interactions in small‐worldness were confined to the delta and beta bands. Beta oscillations are implicated in sensorimotor integration and attention (69–71), whereas delta rhythms contribute to long-term memory formation, speech processing, and comprehension (73–75). Because each oscillatory band supports distinct neural computations and network configurations, these functional specializations likely underlie why clustering coefficient and small‐worldness effects appeared in different bands rather than uniformly across all frequencies.

Despite offering valuable insights, this study has methodological limitations. First, the exclusion rate was relatively high; 41% and 50% of children in the ASD and TD groups, respectively, were excluded due to excessive motion artifacts during MEG acquisition or incomplete MRI scans. These attrition rates likely reflect the inherent difficulty of obtaining high-quality neuroimaging data from young children, especially in clinical populations. Although we believe these exclusions were primarily procedural rather than systematic, they may nonetheless limit the generalizability of our findings to the broader population of children with and without ASD. Second, the TD group was defined based on parental reports and the absence of formal clinical diagnoses. While none of the TD participants were reported to have behavioral or language difficulties, we did not use standardized instruments to screen for subclinical neurodevelopmental or psychiatric traits. Thus, we cannot fully exclude the presence of subtle characteristics that may influence brain network organization in this group. Third, although we excluded participants with known comorbidities or current medication use, we did not systematically assess prior medication history or independently verify the comorbidity status beyond referral diagnosis (ASD group) or parental report (TD group). This may introduce residual confounding effects on the functional connectivity and graph-theoretical metrics reported in this study. Fourth, all participants were of Japanese ethnicity, which may limit the generalizability of our findings to more diverse populations. Future studies including participants from varied racial and ethnic backgrounds are needed to assess the broader applicability of these results. Fifth, the study did not include objective measures of participant vigilance during MEG acquisition. Although no children showed overt signs of drowsiness based on visual inspection of MEG waveforms, this evaluation—conducted by a single author—was subjective and may not have detected brief or subtle fluctuations in alertness. Changes in vigilance are a well-known challenge in resting-state paradigms (81, 82), particularly in pediatric populations, and can affect both spectral power (83) and functional connectivity, especially when using coherence-based measures (84). While PLI-based connectivity and network topology are considered more robust against drowsiness-related confounds (85, 86), the lack of objective vigilance monitoring (e.g., electrooculogram, eye tracking, or behavioral probes) remains a methodological limitation. Future studies should incorporate such measures to enhance the reliability and interpretability of resting-state MEG findings. Sixth, we employed the Desikan–Killiany atlas for cortical parcellation, which is based on anatomical landmarks. While this atlas provides a standardized framework that facilitates comparison across studies, it may not align perfectly with the brain’s functional architecture. Several studies have reported both convergence (87, 88) and divergence (89, 90) between structural and functional boundaries. As such, using anatomically defined parcellations may introduce misalignment between functional activity and regional boundaries. Future work may benefit from using functionally derived or individualized parcellations to better capture the brain’s true network structure. Seventh, we used an identity matrix as the noise covariance in our wMNE for source reconstruction. This approach assumes homoskedastic, uncorrelated noise across sensors and is commonly applied when empirical noise recordings (e.g., empty-room data) are unavailable. However, it does not account for sensor-specific noise variance or spatial correlations, which can reduce source localization accuracy and introduce spatial bias. We recommend that future studies collect same-day baseline or empty-room data to estimate a full noise covariance matrix. Eighth, the wMNE method itself introduces a known spatial bias toward superficial sources (91). Although we applied standard depth weighting and anatomical constraints, these corrections cannot fully eliminate this bias. This may reduce the accuracy of source estimates for deeper cortical or subcortical regions and may influence both connectivity estimates and graph-theoretical metrics. Ninth, while various source reconstruction algorithms are available, no consensus exists regarding the optimal method for resting-state MEG. Each algorithm has specific trade-offs. Compared to alternatives such as beamformers or sLORETA, wMNE offers better control of signal leakage but reduced localization precision (92). We selected wMNE because of its robustness in estimating distributed activity patterns, which aligns with the goals of analyzing resting-state functional connectivity. Nonetheless, this choice entails compromises that should be considered when interpreting the findings. Tenth, the final sample size (23 children in the ASD group, 31 children in the TD group) following participant exclusion represents a limitation that may affect the reliability of our results. As noted by Ioannidis (93, 94), underpowered studies are more likely to miss true effects, overestimate effect sizes when findings are significant, and yield results with lower replicability. In our study, these concerns are particularly relevant to the effects of diagnosis-by-condition interaction in the delta and beta bands, which also informed downstream correlational analyses. Although the interactions reached significance, they should be considered preliminary and interpreted with caution. Future replication with larger, well-powered samples is essential. Eleventh, we occasionally refer to results that did not meet our prespecified significance threshold (e.g., *p* < 0.01 for primary analyses). While such results are explicitly labeled as “exploratory trends” and interpreted cautiously, we acknowledge that relying on near-significant findings increases the risk of Type I errors. Although recent methodological literature [e.g., (95–98)] cautions against rigid dichotomization of statistical outcomes, this remains a debated issue. We have aimed to report exact *p*-values transparently while clearly labeling results that require cautious interpretation. Nonetheless, the use of trends to justify *post hoc* analyses is a limitation of our approach and highlights the need for future confirmatory work. Twelfth, our findings were compared with those of other graph-theoretical studies that varied in connectivity metrics (lagged linear coherence, synchronization likelihood, phase lag index), and graph construction (binary vs. weighted; undirected vs. directed). These discrepancies extend to the number of regions of interest (nodes), which can affect graph metrics, particularly when the node counts fall below 200 (28). Currently, there is no consensus on the optimal methodology for addressing these variations. Nonetheless, despite these methodological differences across studies, many results converge on the notion of atypical small-world properties in children with ASD. Thirteenth, although the Silenz pulse sequence may yield a slightly lower spatial resolution than standard MRI protocols (due to thicker slices and a smaller matrix size), it was appropriate for our objectives. Our priority was to obtain adequate anatomical references while ensuring participant compliance and minimizing motion artifacts. We also implemented quality assurance procedures to confirm that the images met the necessary standards for subsequent analyses. Fourteenth, all the participants with ASD in this study were high-functioning children who were able to remain still during the MEG recordings. Thus, our findings may not be generalizable to children with lower verbal or intellectual abilities, or those who struggle to remain motionless during scanning. Taken together, these considerations highlight the need for careful study design and interpretation in graph-theoretical research, particularly in pediatric ASD populations. Finally, while the pipeline used in this study—constructing binary graphs from PLI-based connectivity—is widely used in pediatric MEG research, it entails certain trade-offs. In particular, applying proportional thresholds enforces equal density across groups and conditions, potentially masking true differences in global connectivity strength (28). For instance, groups with overall weaker but still structured connectivity may lose meaningful connections during thresholding, thereby distorting network topology. Although our threshold-sensitivity analysis (10–30%) confirmed that our findings were stable across a range of thresholds, future work may benefit from weighted-graph approaches that retain continuous PLI values and avoid this limitation.

In conclusion, our study demonstrates that transitioning from DR to EO conditions elicits distinct changes in the functional brain network organization in children with and without ASD, as assessed using key graph-theoretical measures. Notably, the clustering coefficients in the alpha band were higher under EO for both groups, but SW in the delta and beta bands showed diagnosis-by-condition interactions. Children with ASD tended to exhibit greater increases in SW under EO, whereas TD children displayed the opposite pattern. Moreover, the magnitude of these changes correlated with the severity of autistic traits, indicating a potential link between network reactivity and the core features of ASD. Taken together, these findings suggest that age-related and diagnosis-specific mechanisms influence baseline network organization and reactivity to sensory input. Despite these insights, methodological constraints such as a relatively small sample size, variations in connectivity metrics and graph construction, and the inclusion of only high-functioning children with ASD limit the generalizability of our conclusions. Future research should incorporate larger and more diverse samples to improve generalizability, establish standardized protocols for graph-theoretical analyses, and explore baseline-dependent reactivity in multiple frequency bands. By addressing these gaps, we can refine our understanding of functional brain network responses in ASD and potentially identify novel targets for clinical interventions.

## Data Availability

The raw data supporting the conclusions of this article will be made available by the authors, without undue reservation.
