# Supplementary material for: Baseline-dependent network reactivity to visual input in children with autism spectrum disorder: a magnetoencephalography study
Source: Front Psychiatry. 2025 Jul 16;16:1600973. doi: 10.3389/fpsyt.2025.1600973 (PMC12308501; doi:10.3389/fpsyt.2025.1600973)
Supplement: Supplementary file 1 [file SupplementaryFile1.docx]

Supplementary Material

# Diagnostics for mixed-effects models (refers to Results, Section 3.2)

To evaluate key assumptions of our mixed-effects regression models, we generated diagnostic plots for each model central to our primary analyses. As an illustrative example, we describe the diagnostics for the linear mixed-effects model predicting the clustering coefficient (C) in the alpha band. This model included fixed effects for diagnosis (autism spectrum disorder [ASD] vs. typically developing children [TD]), experimental condition (dark room [DR] vs. eyes open [EO]), their interaction, age, and sex, along with a random intercept for each participant to account for within-subject correlations. The same diagnostic procedures were applied to all other primary models:

1. Residual distribution: We plotted histograms of raw residuals overlaid with a normal density curve. This visualization aids in evaluating the normality assumption by highlighting any skewness or kurtosis (Supplementary Figure S1A).

2. Homoscedasticity: To evaluate the assumption of constant variance, we plotted raw residuals against fitted values. The absence of a funnel-shaped pattern in these scatterplots suggests homoscedasticity (Supplementary Figure S1B).

3. Random effects distribution: We examined the distribution of estimated random intercepts for each subject by plotting histograms overlaid with a normal density curve to verify the normality assumption (Supplementary Figure S1C),

Analogous diagnostic plots are provided in Supplementary Figures S2–S4 for the other mixed-effects models, including group-specific analyses. Additional diagnostic panels are available upon request.

**SUPPLEMENTARY FIGURE S1**

Diagnostic checks for the mixed-effects model predicting alpha-band clustering coefficients. (**A**) Histogram of raw residuals (yellow bars) with a superimposed normal density curve (solid green line), indicating approximate normality without pronounced skewness or heavy tails. (**B**) Scatterplot of raw residuals versus fitted values (blue circles) with a horizontal dashed line at zero. The absence of a funnel-shaped pattern suggests roughly constant residual variance across the range of predicted values, supporting homoscedasticity. (**C**) Histogram of estimated random intercepts (per subject) with an overlaid normal density curve (solid green line), indicating approximate normality without pronounced skewness or heavy tails.

Analogous diagnostic plots (residual histogram, residuals vs. fitted values, and random intercept histogram) are provided in Supplementary Figures S2–S4 for each additional mixed-effects model central to our analyses. Additional panels not shown here are available upon request.

**SUPPLEMENTARY FIGURE S2**

Diagnostics for the mixed-effects models predicting delta- and beta-band SW, including both the full sample (upper row) and group-specific analyses for TD (middle row) and ASD (lower row) groups. (**A-δ, A-β**) Histograms of raw residuals (yellow bars) for delta-band and beta-band SW, respectively, with overlaid normal density curves. (**A-δ-ASD, A-δ-TD**) Histograms of raw residuals for delta-band SW in ASD and TD groups, respectively. (**A-β-ASD, A-β-TD**) Histograms of raw residuals for beta-band SW in ASD and TD groups, respectively. (**B-δ, B-β**) Scatterplots of raw residuals versus fitted values (blue circles) for delta-band and beta-band SW, respectively, with horizontal dashed lines at zero. (**B-δ-ASD, B-δ-TD**) Scatterplots of raw residuals versus fitted values for delta-band SW in ASD and TD groups, respectively. (**B-β-ASD, B-β-TD**) Scatterplots of raw residuals versus fitted values for beta-band SW in ASD and TD groups, respectively. (**C-δ, C-β**) Histograms of estimated random intercepts (per subject) for delta-band and beta-band SW, respectively, with overlaid normal density curves. (**C-δ-ASD, C-δ-TD**) Histograms of estimated random intercepts for delta-band SW in ASD and TD groups, respectively. (**C-β-ASD, C-β-TD**) Histograms of estimated random intercepts for beta-band SW in ASD and TD groups, respectively. *Abbreviations:* ASD, autism spectrum disorder; TD, typically developing children; SW, small-worldness.

These diagnostic plots collectively support the validity of our mixed-effects modeling approach by confirming the residual normality and homoscedasticity across models.

# Diagnostics for linear regression between EO-induced SW changes and autistic traits (refers to Results, Section 3.3)

We conducted diagnostic checks for the linear regression models examining the relationship between EO-induced changes in small-worldness (ΔSW) and autistic traits, as measured by raw total SRS scores. Each model included predictors for ΔSW between EO and DR conditions (in either the delta or beta band), diagnosis (ASD vs. TD), the interaction between ΔSW and diagnosis, age, and sex. Group-specific models (ASD-only or TD-only) included ΔSW, age, and sex as predictors.

To evaluate the assumptions of linear regression, we generated the following diagnostic plots:

1. Residual distribution: Histograms of raw residuals overlaid with normal density curves were used to assess residual normality, focusing on symmetry and the presence of skewness or kurtosis.

2. Homoscedasticity: Residuals were plotted against fitted values to assess variance constancy. In the model for delta-band SW, the residuals exhibited a funnel-shaped pattern with increasing spread at higher predicted values (Supplementary Figure S3B–δ) indicating heteroscedasticity (Breusch–Pagan test, χ^2^(1) =11.59, *p* < 0.001). Due to this, and to ensure consistency across all related models, we employed heteroscedasticity-robust standard errors throughout (1).

**SUPPLEMENTARY FIGURE S3**

Diagnostic checks for linear regression models predicting raw total SRS scores based on EO-induced ΔSW. (**A–δ, A–β**) Histograms of raw residuals for delta and beta-band models (yellow bars), with overlaid normal density curves. (**A–δ–ASD, A–δ–TD**) Residual histograms for delta-band models in ASD and TD groups. (**A–β–ASD, A–β–TD**) Residual histograms for beta-band models in ASD and TD groups. (**B–δ, B–β**) Scatterplots of residuals versus fitted values for delta- and beta-band full-sample models. B–δ shows a funnel-shaped pattern indicating increasing residual spread with higher predicted values, suggesting heteroscedasticity (Breusch–Pagan test: χ²(1) = 11.59, *p* < 0.001). (**B–δ–ASD, B–δ–TD**) Scatterplots of residuals vs. fitted values for delta-band subgroup models. B–δ–TD similarly indicates heteroscedasticity (Breusch–Pagan test: χ²(1) = 6.28, *p* = 0.012). (**B–β–ASD, B–β–TD**) Scatterplots of residuals vs. fitted values for beta-band subgroup models. *Abbreviations:* SRS, Social Responsiveness Scale; EO, eyes open; ΔSW, change in small-worldness; ASD, autism spectrum disorder; TD, typically developing children.

These diagnostics highlight heteroscedasticity concerns in some cases, warranting the use of robust standard errors for reliable inference.

# Diagnostics for linear regression between EO-induced changes and baseline measures (refers to Results, section 3.4)

In Section 3.4, we examined whether EO-induced changes in graph metrics (EO − DR) could be predicted by their baseline values during the DR condition. Separate linear regression models were estimated for each frequency band and graph measure. Each model included predictors for baseline metric (DR), diagnosis (ASD vs. TD), their interaction, age, and sex.

We applied the same diagnostic procedures described above to evaluate model assumptions.

1. Residual distribution: Histograms of raw residuals overlaid with a normal density curve were used to assess the normality of residuals visually.

2. Homoscedasticity: Residuals were plotted against fitted values to evaluate variance constancy. The absence of a funnel-like pattern suggests homoscedasticity.

**SUPPLEMENTARY FIGURE S4**

Diagnostic checks for linear regression models predicting EO-induced changes in graph metrics based on their baseline (DR) values. (**A-x-u**) Histograms of raw residuals for frequency band x and graph measure u. For example, “A-δ-SW” is the histogram of raw residuals for the delta-band SW model. The overlaid normal density curve (solid line) highlights whether the residuals approximate a Gaussian distribution. (**B-x-u**) Scatterplots of raw residuals versus fitted values for frequency band x and graph measure u. For example, “B-δ-SW” is the residuals-vs-fitted plot for the delta-band SW model. A lack of a funnel-shaped pattern indicates approximately constant variance (homoscedasticity). *Abbreviations:* EO, eyes open; DR, dark room; SW, small-worldness.

These plots provide visual confirmation that model assumptions were generally met across frequency bands and graph measures.

# Threshold variability

To ensure that the binary‐graph findings were not idiosyncratically driven by a specific choice of network density, we recomputed the clustering coefficient (C), characteristic path length (L), and small‐worldness (SW) across proportional thresholds ranging from 10% to 30%, in 2% increments. These metrics were computed following the same procedures outlined in the main text.

The following supplementary tables support the robustness of our key findings across a plausible range of graph densities.

**TABLES S1–S10** Effects of diagnosis, experimental condition, and their interaction on graph metrics (C, L, SW) at proportional thresholds of 10%, 12%, 14%, 16%, 18%, 22%, 24%, 26%, 28%, and 30%, respectively.

**TABLES S11–S20** Relationships between EO-induced changes (EO vs. EC) and autistic traits at the same set of thresholds.

**TABLES S21–S30** Effects of baseline graph measures (C, L, SW) on EO-induced changes at each threshold.

# Reference

1. White H. A heteroskedasticity-consistent covariance matrix estimator and a direct test for heteroskedasticity. *Econometrica.* (1980) 48:817–38. doi: 10.2307/1912934
