# Supplementary figures and images for: Baseline-dependent network reactivity to visual input in children with autism spectrum disorder: a magnetoencephalography study
Source: Front Psychiatry. 2025 Jul 16;16:1600973. doi: 10.3389/fpsyt.2025.1600973 (PMC12308501; doi:10.3389/fpsyt.2025.1600973)

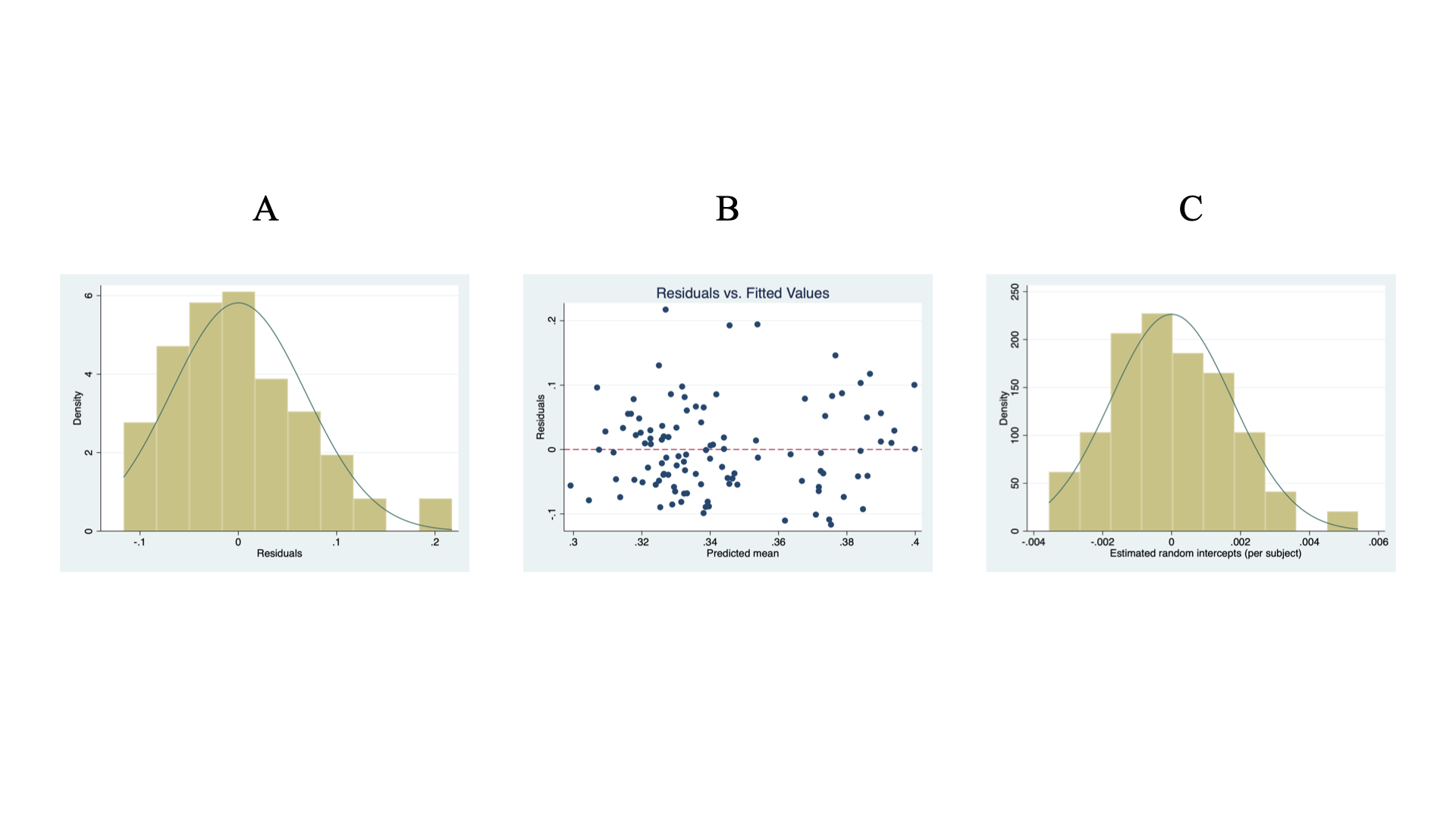

Supplement: Supplementary file 2 [file Image1.tiff]

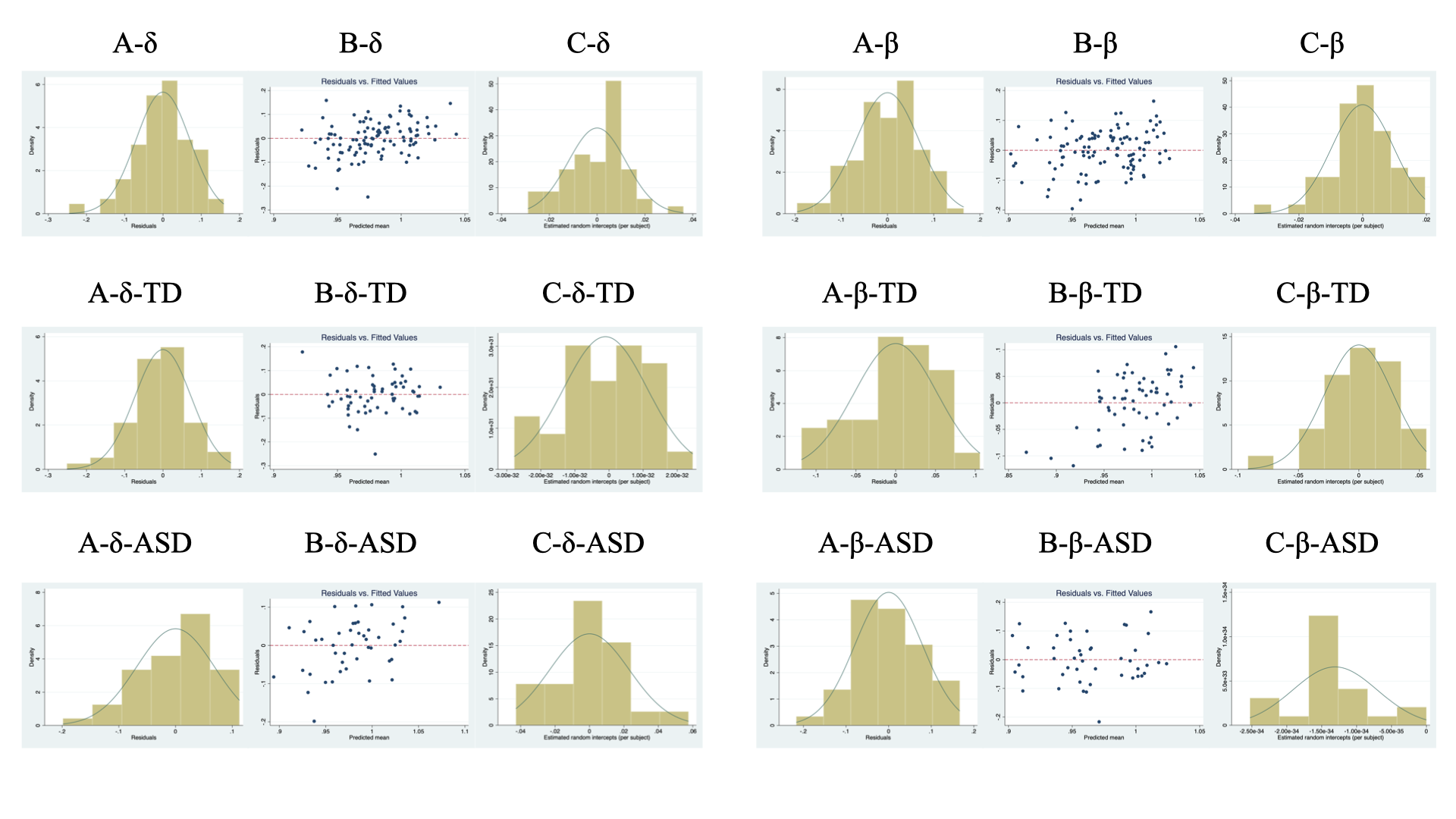

Supplement: Supplementary file 3 [file Image2.tiff]

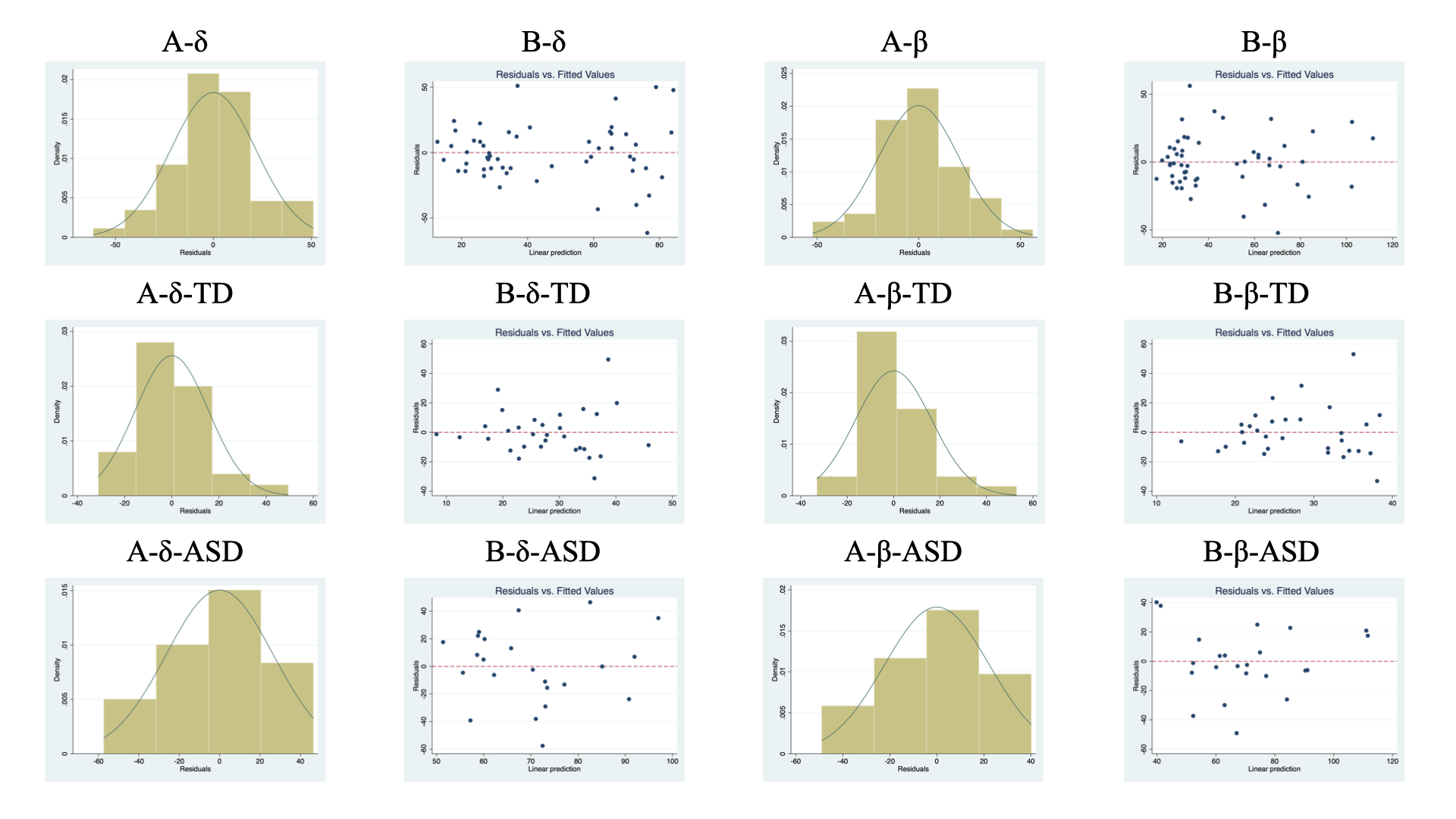

Supplement: Supplementary file 4 [file Image3.tiff]

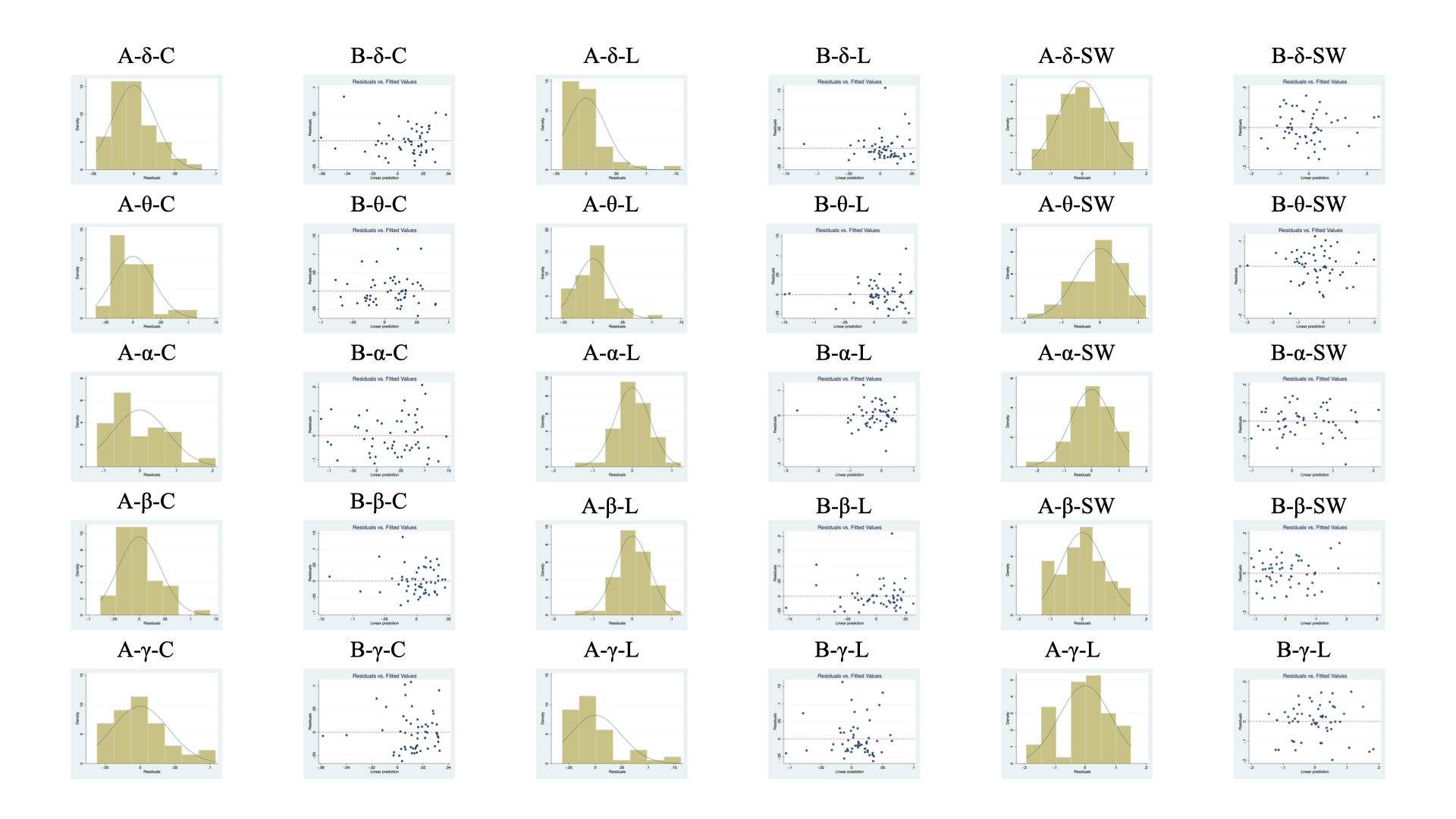

Supplement: Supplementary file 5 [file Image4.tiff]
